# Preparation and Reaction Chemistry of Novel Silicon-Substituted 1,3-Dienes

**DOI:** 10.3390/molecules200916892

**Published:** 2015-09-16

**Authors:** Partha P. Choudhury, Mark E. Welker

**Affiliations:** Department of Chemistry, Wake Forest University, P. O. Box 7486, Winston-Salem, NC 27109, USA; E-Mail: pchoudhury@gru.edu

**Keywords:** organosilanes, 1,3-dienes, enyne metathesis, Diels-Alder, cross coupling

## Abstract

2-Silicon-substituted 1,3-dienes containing non transferrable groups known to promote transmetallation were prepared by Grignard chemistry and enyne metathesis. These dienes participated in one pot metathesis/Diels-Alder reactions in regio- and diastereoselective fashions. Electron-rich alkenes showed the fastest rates in metathesis reactions, and ethylene, a commonly used metathesis promoter slowed enyne metathesis. 2-Pyridyldimethylsilyl and 2-thienyldimethylsilyl substituted Diels-Alder cycloadducts participated in cross-coupling chemistry and the 2-thienyldimethylsilyl substituted cycloadducts underwent cross-coupling under very mild reaction conditions.

## 1. Introduction

We have been interested in the preparation and reaction chemistry of metal-substituted dienes for over 20 years. Initially, we prepared a number of transition metal-substituted dienes [1,2] for these studies but, more recently, we have been interested in the investigation of silicon- and boron-substituted dienes [3,4,5,6,7]. We have reported the preparation of 2-silicon-substituted 1,3-butadienes by a variety of synthetic routes and demonstrated that they could be used in sequential Diels-Alder/cross-coupling reactions [8,9,10,11,12,13]. Here we report the preparation of new 2-silicon-substituted 1,3-dienes containing silicon substituents known to promote transmetallation (hence, the ability to participate in cross-coupling reactions under very mild conditions) and their Diels-Alder/cross-coupling reactions. While the new chemistry disclosed here was not aimed specifically at *cis*-clerodane synthesis targets, we believe it can also be used to access those biologically-significant core structures [14].

## 2. Results and Discussion

### 2.1. 2-Silicon-Substituted Diene Synthesis via Grignard Chemistry

**Scheme 1:**
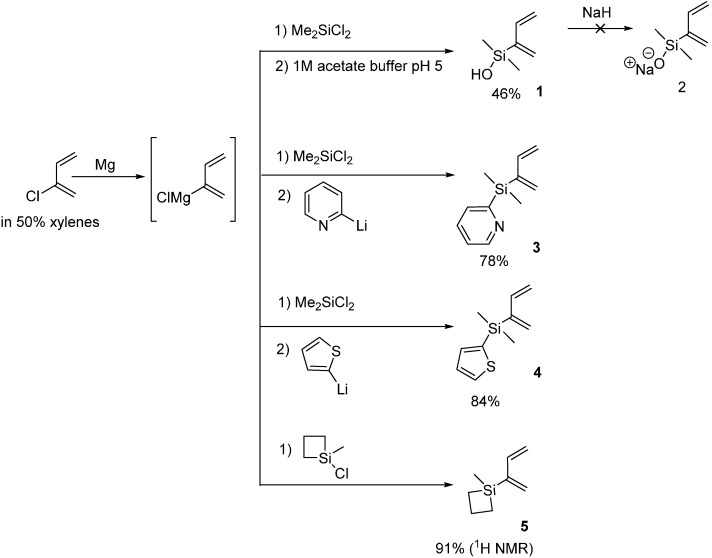
Reactions of the Grignard reagent from chloroprene with silyl chlorides.

In 2014, we first reported the preparation and isolation of buta-1,3-dien-2-yldimethylsilanol (**1**) [13], however, this molecule proved to be very unstable towards dimerization, so we began to look for alternative silicon-substituted dienes with higher stability but also containing silicon substituents known to promote transmetallation. In 1999, the Denmark group reported silacyclobutanes as masked silanol equivalents [15] and this was followed by reports from the Itami group [16] that the 2-pyridyldimethylsilyl group and from the Hiyama group that the 2-thienyldimethyl silyl group [17] could similarly function as masked silanol precursors. In 2007, we first reported synthesis of 2-silicon-substituted 1,3-butadienes from chloroprene via zinc catalyzed Grignard reactions with the corresponding halo silanes [9]. In the present work, we used that same strategy and successfully prepared the 2-pyridyldimethylsilyl (**3**) and 2-thienyldimethylsilyl (**4**)-substituted dienes (Scheme 1). Siletane diene (**5**) was also prepared by this route but we could not completely separate it from xylene by distillation or chromatography so we could not completely characterize it.

### 2.2. Diels-Alder/Cross-Coupling Reactions

**Scheme 2:**
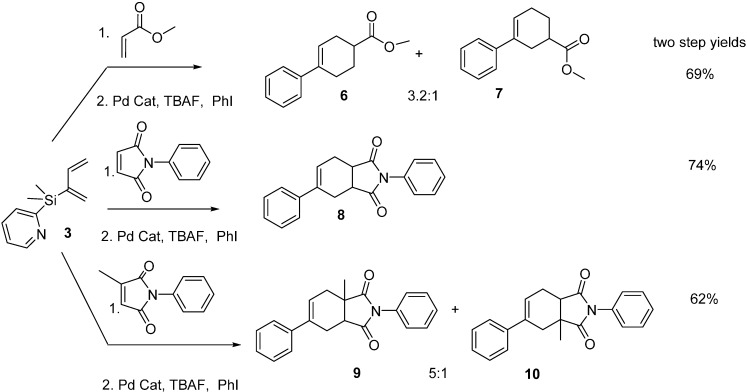
Diels-Alder/cross-coupling reactions of 2-pyridyldimethylsilyl-1,3-butadiene.

**Scheme 3:**
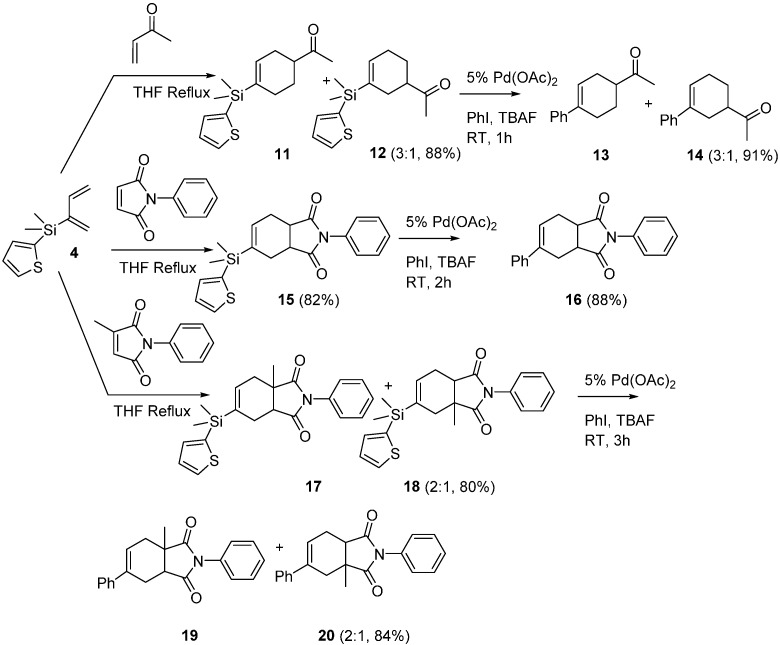
Diels-Alder reactions of 2-thienyldimethylsilyl-1,3-butadiene.

We first monitored the reactivity of dienes **3** and **4** with *N*-phenylmaleimide under pseudo-first order reaction conditions at 25 °C in CDCl_3_ by ^1^H-NMR, and calculated *t*_1/2*’s*_ of 22 min and 29 min, respectively. These 2 dienes are not quite as reactive as the silatrane diene we reported back in 2007 (*t*_1/2_ of 18 min at 0 °C) but they are very similar in reactivity to Danishefsky’s diene [9]. We then took diene **3** and reacted it with three types of dienophiles (methyl acrylate, *N*-phenylmaleimide, and *N*-phenyl citraconimide) followed by Pd (II) catalyst, tetra-*n*-butylammonium fluoride (TBAF) and aryl halide in one-pot sequential Diels-Alder/cross-coupling reactions (Scheme 2). The unsymmetrical dienophiles produced predominantly *para* products (**6**, **9**) and yields for the two step process ranged from 62%–74% (avg per step 79%–87%). The 2-thienyldimethylsilyl diene **4** also reacted with these same dienophiles and cycloadducts (**11**, **12**, **15**, **17**, **18**) were again obtained in good yields, albeit with slightly lower regioselectivities than observed with diene **3** for the unsymmetrical dienophiles (Scheme 3). However, we were initially not able to produce cross-coupled cycloadducts from these Diels-Alder products (**11**, **12**, **15**, **17**, **18**) using conditions which worked for the 2-pyridyldimethylsilyl cycloadducts (mild heating to 50 °C just lead to cycloadduct decomposition). Cross-coupling reactions performed under even milder conditions (25 °C for 1–3 h) did produce the desired cross-coupled products (**13**, **14**, **16**, **19**, **20**) in excellent yields (Scheme 3). These Diels-Alder products (**11**, **12**, **15**, **17**, **18**) are the first silicon-substituted cyclohexenes that we are aware of that would participate in cross-coupling reactions at 25 °C.

### 2.3. 2-Silicon-Substituted Diene Synthesis via Enyne Metathesis

**Scheme 4:**
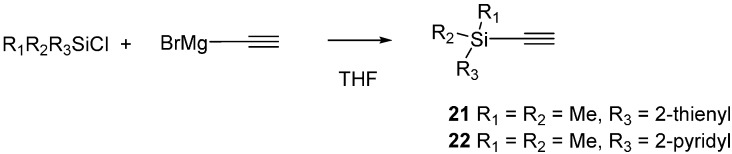
Preparation of silyl alkynes.

In 2010 we published a methodology based on enyne metathesis to prepare 4-aryl- and 4-alkyl-2-silyl-1,3-butadienes [11]. Pietraszuk and co-workers also recently reported a related cross-metathesis protocol for silyl alkynes [18]. In the present work, we also wanted to synthesize more highly-substituted silicon dienes containing nontransferable groups known to promote transmetallation. Thus, to investigate that possibility we first prepared both 2-thienyldimethylsilyl- and 2-pyridinyldimethylsilylethyne (**21**,**22**) (Scheme 4). These compounds were prepared as described by Denmark via addition of ethynylmagnesium bromide to the appropriate silyl chloride [19].

**Scheme 5:**
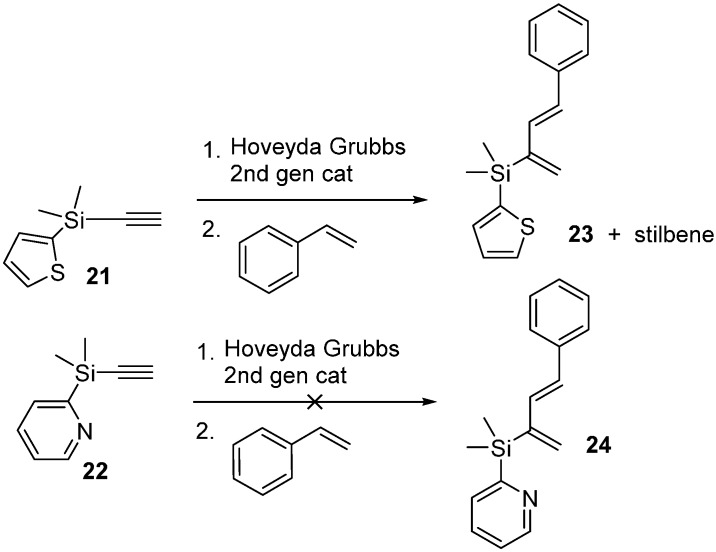
Initial attempts at enyne metathesis of **21** and **22**.

We initially investigated metathesis of these two alkynes with styrene using Hoveyda Grubbs 2nd generation catalyst and the 5:1 alkene:alkyne ratio we had used originally in 2010 [11]. Under these conditions, the 2-pyridyldimethylsilyl alkyne produced no observable diene and the 2-thienyldimethylsilyl alkyne produced a diene, but it was very difficult to separate it from the stilbene (alkene metathesis) byproduct. We increased the amount of ruthenium complex used all the way up to a stoichiometric amount with alkyne **22** and saw no diene, so we suspect the pyridine group prevents the desired metathesis (Scheme 5). We also attempted to prepare a number of other silyl alkynes with the procedure used to prepare **21** and **22** (R_1_ = R_2_ = Me, R_3_ = Cl; R_1_ = R_2_ = iPr, R_3_ = H; R_1_ = R_2_ = -CH_2_CH_2_CH_2_-, R_3_ = Me) but in these other cases those alkynes decomposed upon attempted purification. Since the alkyne decomposition appeared to occur when the extraction solutions were concentrated, we also attempted using these additional alkynes in cross-metathesis without purification, but this also proved unsuccessful.

**Scheme 6:**
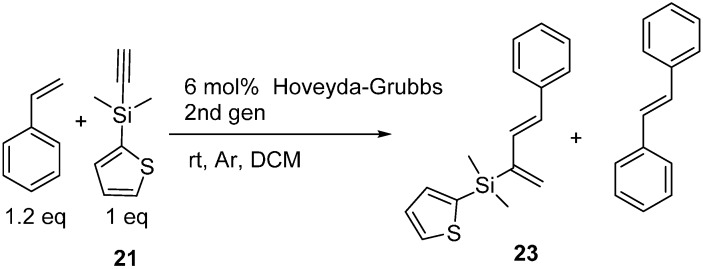
Optimized enyne metathesis.

Olefin metathesis competes with enyne cross-metathesis and we found it impossible to completely separate **23** by chromatography or distillation from the large amounts of byproduct stilbene being produced when excess styrene was used. To suppress olefin metathesis we attempted limiting alkene concentrations similar to the work that Clark and Diver had reported in 2011 [20]. We are able to drive the reaction to completion (as judged by ^1^H-NMR disappearance of the alkyne proton in **21**) with styrene with 6 mol % Hoveyda Grubbs 2nd generation catalyst loading at an alkene:alkyne ratio of 1.2:1 (Scheme 6). Grubbs 1st and 2nd generation catalysts, Hoveyda Grubbs 1st generation and the Zhan 1B catalyst were also screened at 6% loadings and none proved superior to Hoveyda Grubbs second generation catalyst for this cross metathesis.

While we were able to minimize stilbene production in the preparation of **23**, we were not able to isolate **23** analytically pure by distillation or chromatography. In our previous work with benzyl(ethynyl)dimethylsilane we had performed sequential metathesis/Diels-Alder chemistry without isolating dienes so we did not view this as an insurmountable synthetic problem [11,12].

**Scheme 7:**
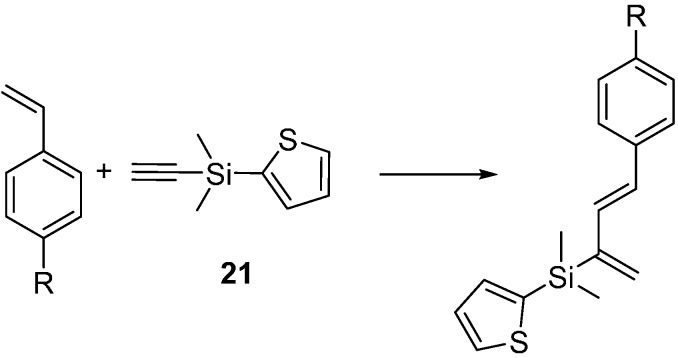
Enyne metathesis of **21**.

We next moved to an investigation of cross-metathesis using styrenes that contained both electron donating and withdrawing groups. The metathesis chemistry of the 2-thienyldimethylsilylethyne (**21**) proved very different from the trends observed by Diver’s group [21] and very different from what we had observed previously with dimethylbenzylsilylethyne [11]. The reaction of **21** with *p*-vinylanisole was complete with 3 mol % catalyst within 2 h under reflux conditions, whereas *p*-chlorostyrene needed 6 mol % catalyst and was refluxed for 30 h (Scheme 7, Table 1). As mentioned above, Diver’s group had studied the effect of alkene electronics with Grubbs second generation catalyst and their work revealed moderate electron withdrawing groups on the phenyl ring facilitated enyne metathesis (*p*-methoxystyrene was approximately two orders of magnitude slower than *p*-bromostyrene), and that these reactions went via an arylidene first mechanism [21]. Our results here showed the opposite trend, *i.e*., the moderately electron-withdrawing chlorostyrene was least reactive, but these reactions were performed using Hoveyda Grubbs second generation catalyst rather than the Grubbs second generation catalyst Diver used. Completion of these reactions was judged by disappearance of the alkyne proton of **21** by ^1^H-NMR as described above. 

**Table 1:** Enyne Metathesis Conditions.

R	Catalyst Loading (mol %)	Temperature	Time (Disappearance of the Alkyne Peak in ^1^H-NMR)
-OCH_3_	7	RT	3 h
-OCH_3_	3	reflux	2 h
-H	6	reflux	12 h
-Cl	6	reflux	30 h

Ethylene has been reported by researchers [22,23] as a promoter of metathesis until recently Gregg, Keister, and Diver reported on the inhibitory effect of excess ethylene in enyne metathesis [24]. These reactions which were inhibited by ethylene proceed via a mechanism where there is a ruthenacyclobutane catalyst resting state and this ruthenacyclobutane is formed via reaction of ruthenium methylidene with ethylene. When we attempted the enyne metathesis reactions reported here in the presence of one atmosphere of ethylene, we also noted metathesis inhibition. Whereas p-methoxystyrene had reacted completely with the silyl alkyne (**21**) in 3 h at room temperature in the absence of ethylene, we noted only ~50% conversion under ethylene; similarly, styrene after 12 h of reflux under ethylene showed only about 30% conversion. 

### 2.4. Tandem Methathesis/Diels-Alder Reactions and Subsequent Cross-Coupling

**Scheme 8:**
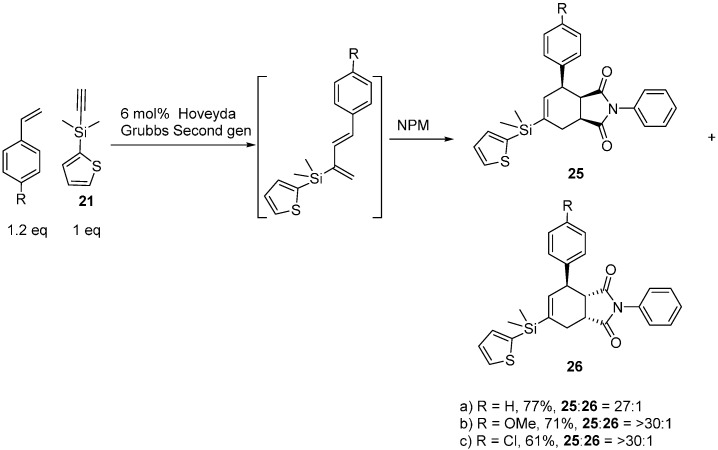
One-pot enyne metathesis/Diels-Alder reactions.

**Scheme 9:**
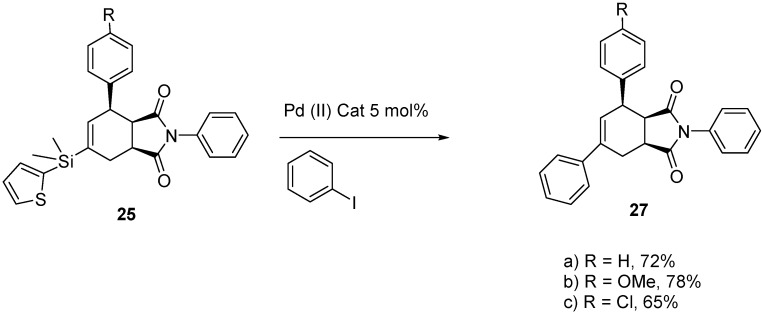
Cross-coupling of aryl substituted 2-thienyldimethylsilyl-substituted cycloadducts.

Once we optimized reaction conditions, we carried out one-pot methatheses and Diels-Alder reactions (Scheme 8). All three dienes reacted in highly regio*-*, and diastereo-selective fashions. We did not observe any meta Diels-Alder adduct and, in the case of **25a** and **26a**, the observed syn:anti ratio was 27:1. The *syn* and *anti* diastereomers (**25a** and **26a**) were separated and analyzed using NOESY data (Supplementary Material) to establish their stereochemical assignments. When we analyzed crude reaction mixtures of tandem metathesis and Diels-Alder products by ^1^H-NMR spectroscopy we did not observe any *exo* adduct formation for *p*-chlorostyerene and *p-*vinylanisole. Upon purification on silica, we exclusively obtained the *endo* adducts (**25b** and **25c**). All of these silicon-substituted cycloadducts (**25a**–**c**) also then participated in cross-coupling reactions with iodobenzene at room temperature to produce cycloadducts (**27**) (Scheme 9). While the new chemistry disclosed here was not aimed specifically at *cis*-clerodane targets, we believe it can also be used to access those biologically-significant core structures [14].

## 3. Experimental Section

### 3.1. General Information

The proton nuclear magnetic resonance (^1^H-NMR) spectra were obtained using a Bruker Avance 300 MHz spectrometer operating at 300.1 MHz or a Bruker Avance 500 MHz spectrometer operating at 500.1 MHz. ^13^C-NMR spectra were obtained using a Bruker Avance 300 MHz spectrometer operating at 75.5 MHz. ^1^H- and ^13^C-NMR spectra were referenced to the residual proton or carbon signals of the respective deuterated solvents. All elemental analyses were performed by Atlantic Microlabs Inc., Norcross, GA, USA. High-resolution mass spectrometry was performed at the UNC Mass Spectrometry Facility, Chapel Hill, NC, USA or the Northwestern University Mass Spectrometry facility.

All reactions were carried out under an atmosphere of nitrogen. Tetrahydrofuran (THF) was degassed with argon and then passed through two 4 × 36 inch columns of anhydrous neutral A-2 alumina (8 × 14 mesh; activated under a flow of Ar at 350 °C for 3 h) to remove water. Deuterated solvents were purchased from Cambridge Isotope Laboratories (Tewksbury, MA, USA) and dried over molecular sieves. Sodium sulfate, sodium hydroxide, magnesium small turnings, and 1,2-dibromoethane were purchased from Sigma-Aldrich Chemical Company (St. Louis, MO, USA) and used as received. 2-Chloro-1,3-butadiene, 50% in xylene (chloroprene) was purchased from Pfaltz & Bauer, Inc. (Waterbury, CT, USA) and used as received.

### 3.2. Silyl Diene Synthesis via Grignard Chemistry

#### 3.2.1. 2-(Buta-1,3-dien-2-yldimethylsilyl)pyridine **3**

A 100 mL flame-dried two-neck round-bottom flask was equipped with a magnetic stir bar, addition funnel, and reflux condenser under Ar. Mg turnings (1.00 g, 41.66 mmol) were added, followed by anhydrous THF (5 mL), followed by 1,2-dibromoethane (266 µL, 3.1 mmol). Activation of Mg was confirmed by the evolution of ethane gas. Anhydrous ZnCl_2_ (0.168 g, 1.23 mmol) was dissolved in THF (3 mL) and was added to the reaction mixture. After stirring for 5 min the color of the solution became milky white. Anhydrous THF (20 mL) was added and it was set to reflux under Ar. After refluxing for 30 min chloroprene in 50% xylenes (5.04 mL, 26 mmol) was loaded into the addition funnel. 1,2-Dibromoethane (0.533 mL, 6.2 mmol) and THF (5 mL) were mixed with the chloroprene and it was slowly added over 30 min. Upon completion of the addition, the solution was refluxed for 45 min. The color of the reaction mixture turned green and it was cooled to room temperature. The reaction mixture was cannula transferred to a round bottom flask containing dimethyldichlorosilane (3.00 mL, 25 mmol) and diethyl ether (50 mL) and stirred for 2 h under Ar. In a separate flame-dried one-neck 250 mL flask containing a magnetic stir bar, 2-bromopyridine (2.5 mL, 26 mmol) was added. Anhydrous diethyl ether (15 mL) was added and the flask was sealed with a septum. The entire mixture was kept under positive pressure of Ar and cooled to −78 °C. At the same temperature, *n*-butyllithium (17 mL of a 1.6 M solution in hexanes, 27.3 mmol) was added and was stirred for 30 min. The color changed to orange. To this reaction mixture buta-1,3-dien-2-ylchlorodimethylsilane was cannula transferred at −78 °C. The solution was allowed to reach room temperature and stirred for 12 h. The resultant mixture was diluted with diethyl ether (100 mL), washed with sat NaHCO_3_ solution (20 mL) and 1 M HCl (20 mL). The organic layer was collected and dried over Na_2_SO_4_, concentrated by rotary evaporation and subsequently purified by column chromatography (neutral alumina stationary phase, 50:1 pentane: ethylacetate, R_f_ 0.5). Diene **3** was obtained as a colorless liquid (3.69 g, 19.5 mmol, 78%). ^1^H-NMR (300 MHz, Chloroform-d) δ 8.79 (dt, *J* = 4.9, 1.4 Hz, 1H), 7.66–7.48 (m, 2H), 7.19 (ddd, *J* = 7.1, 4.9, 1.8 Hz, 1H), 6.49 (dd, *J* = 17.7, 10.7 Hz, 1H), 5.92 (d, *J* = 3.0 Hz, 1H), 5.58 (d, *J* = 3.0 Hz, 1H), 5.13 (d, *J* = 17.7 Hz, 1H), 5.02 (d, *J* = 10.7 Hz, 1H), 0.44 (s, 6H); ^13^C-NMR (75 MHz, Chloroform-*d*) δ 166.5, 150.2, 146.8, 141.0, 133.9, 130.7, 129.7, 122.7, 116.8, −2.9; HRMS calcd for C_11_H_16_NSi (M + H)^+^ 190.1052, found 190.1056.

#### 3.2.2. 2-(Buta-1,3-dien-2-yldimethylsilyl)thiophene **4**

A 250 mL two-neck round-bottom flask equipped with magnetic stir bar was flame-dried and cooled under Ar. Mg (3.00 g, 123 mmol) was added and a reflux condenser and addition funnel were attached. The set up was kept under a positive pressure of Ar. THF (10 mL), and 1,2-dibromoethane (0.8 mL, 9.28 mmol) was added the reaction mixture. Activation of Mg was confirmed by the evolution of ethane. ZnCl_2_ (0.504 g, 3.7 mmol) was dissolved in THF (5 mL) and was added to the reaction mixture. After stirring for 5 min the color of the solution became milky white. THF (20 mL) was added and it was set to reflux under Ar. After refluxing for 30 min chloroprene in 50% xylenes (15.1 mL, 78 mmol) was loaded into the addition funnel. 1,2-Dibromoethane (1.6 mL, 18.6 mmol) and THF (15 mL) were mixed with the chloroprene and it was slowly added over 30 min, followed by reflux for 45 min. The color of the reaction mixture turned green and it was cooled to room temperature. In a separate flame-dried one-neck 500 mL flask a magnetic stir bar was added. A rubber septum was attached. Dimethyldichlorosilane (9.1 mL, 75 mmol) was diluted with THF (50 mL) and were added through the septum. The halo diene Grignard reagent was cannula transferred to this solution under Ar and was stirred at room temperature for 2 h. The solution was then cooled to −78 °C and 2-lithiothiophene (75 mL of a 1 M soln in THF/hexanes, 75 mmol) was added drop-wise. After overnight stirring, it was diluted with diethyl ether (100 mL) and the reaction mixture was washed with 1 M HCl (20 mL), saturated NaHCO_3_ (20 mL) solution and brine (20 mL). The organic layer was collected, dried over sodium sulfate, and concentrated using a rotary evaporator. The pure compound was isolated by vacuum distillation at 50 °C at 4 mm Hg (12.2 g, 63 mmol, 84%). ^1^H-NMR (300 MHz, Chloroform-*d*) δ 7.62 (dd, *J* = 4.6, 0.9 Hz, 1H), 7.31 (dd, *J* = 3.3, 1.0 Hz, 1H), 7.19 (dd, *J* = 4.6, 3.3 Hz, 1H), 6.49 (dd, *J* = 17.7, 10.8 Hz, 1H), 5.87 (d, *J* = 2.9 Hz, 1H), 5.53 (d, *J* = 3.0 Hz, 1H), 5.22 (d, *J* = 17.7 Hz, 1H), 5.06 (d, *J* = 10.7 Hz, 1H), 0.50 (s, 6H). ^13^C-NMR (75 MHz, Chloroform-*d*) δ 147.3, 140.7, 137.5, 135.1, 130.9, 130.3, 128.1, 116.6, −1.0; LRMS calcd for C_10_H_15_SSi (M + H)^+^ 195.1, found 195.1.

#### 3.2.3. 1-(Buta-1,3-dien-2-yl)-1-methylsiletane **5**

Chloroprene in 50% xylenes (1.0 mL, 5.15 mmol) and 1-chloro-1-methylsiletane (0.53 mL, 4.3 mmol) were used according to the procedure described above to yield a light yellow colored liquid. ^1^H-NMR of the crude product (1.1 g) indicated formation of the diene (^1^H-NMR diagnostic peaks) (300 MHz, Chloroform-*d*) δ 6.50 (ddt, *J* = 17.7, 10.5, 0.8 Hz, 1H), 5.82 (dt, *J* = 3.1, 0.7 Hz, 1H), 5.57 (ddd, *J* = 3.5, 1.2, 0.6 Hz, 1H), 5.24–5.07 (m, 2H), 1.34–0.86 (m, 6H), 0.42 (s, 3H).

### 3.3. General Procedure of One-Pot Diels-Alder and Cross-Coupling Reactions of ***3***

Diene **3** (1 equiv) was added to dienophile (1.1 equiv) and heated in a round-bottom flask with THF solvent (5–10 mL) for 24 h under Ar. Pd (II) catalyst (5 mol %) was added with aryl halide (1 equiv) and TBAF (1 equiv). The reaction mixture was heated under Ar for 4 h at 60 °C. The reaction mixture was filtered through a silica gel pad (EtOAc) and solvent was removed under reduced pressure. The crude product was purified by column chromatography (dichloromethane mobile phase).

#### 3.3.1. Methyl 2,3,4,5-Tetrahydro-[1,1′-biphenyl]-4-carboxylate **6** and Methyl 2,3,4,5-Tetrahydro-[1,1′-biphenyl]-3-carboxylate **7**

Following the general procedure, diene **3** (0.1 g, 0.53 mmol) and methyl vinyl ketone (0.48 g, 0.69 mmol), PdCl_2_(PhCN)_2_ (0.010 g, 0.026 mmol), iodobenzene (0.108 g, 0.53 mmol), and TBAF (0.53 mL of a 1 M soln in THF, 0.53 mmol) were reacted. The crude product was purified by silica gel flash column chromatography using pure DCM to yield **6** and **7** (R_f_ 0.6, 0.073 g, 0.37 mmol, 69%) identical by ^1^H-NMR comparison to previously reported material.[25] Ratio of para and meta regio-isomers was determined by ^1^H-NMR (3.2:1).

#### 3.3.2. 2,5-Diphenyl-3a,4,7,7a-tetrahydro-1*H*-isoindole-1,3(2*H*)-dione **8**

Following the general procedure diene **3** (0.094 g, 0.5 mmol), *N*-phenylmaleimide (0.094 g, 0.55 mmol), PdCl_2_(PhCN)_2_ (0.010 g, 0.026 mmol), iodobenzene (0.102 g, 0.5 mmol), and TBAF (0.5 mL of a 1 M soln in THF, 0.5 mmol) were used. The crude product was purified by silica gel flash column chromatography using DCM to yield **8** (R_f_ 0.5, 0.112 g, 0.0.37 mmol, 74%), identical by ^1^H-NMR comparison to previously reported material [25]. 

#### 3.3.3. 3a-Methyl-2,6-diphenyl-3a,4,7,7a-tetrahydro-1*H*-isoindole-1,3(2*H*)-dione **9** and 3a-Methyl-2,5-diphenyl-3a,4,7,7a-tetrahydro-1*H*-isoindole-1,3(2*H*)-dione **10**

Following the general procedure diene **3** (0.094 g, 0.5 mmol), 2 methyl-*N*-phenylmaleimide (0.103 g, 0.55 mmol), PdCl_2_(PhCN)_2_ (0.010 g, 0.026 mmol), iodobenzene (0.102 g, 0.5 mmol), and TBAF (0.5 mL of a 1 M soln in THF, 0.5 mmol) in THF solution were used. The crude product was purified by silica gel flash column chromatography using DCM to yield **9** and **10** (R_f_ 0.53, 0.095 g, 0.31 mmol, 62%), identical by ^1^H-NMR comparison to previously reported material [25]. Ratio of *para*:*meta* product was found out to be 5:1 by ^1^H-NMR.

### 3.4. General Procedure of Diels-Alder Reactions of Diene ***4***

Diene **4** (1.2 equiv) was added to a 10 mL pressure tube followed by anhydrous THF (4 mL). Dienophile (1 equiv) was added and degassed for 4 min. The tube was sealed using a crimp cap and the vial was heated as described below.

#### 3.4.1. 1-(4-(Dimethyl(thiophen-2-yl)silyl)cyclohex-3-en-1-yl)ethan-1-one **11** and 1-(3-(Dimethyl(thiophen-2-yl)silyl)cyclohex-3-en-1-yl)ethan-1-one **12**

Diene **4** (1.11 g, 5.72 mmol) and methyl vinyl ketone (0.4 mL, 4.76 mmol) were reacted for 12 h at 66 °C. The solvent was then removed by rotary evaporation and the crude product dried under vacuum. The crude product was purified by silica gel flash column chromatography using DCM as mobile phase (R_f_ 0.6). Compounds **11** and **12** were obtained as a colorless liquid (1.08 g, 4.09 mmol, 88% yield). Ratio of *para*:*meta* isomer was 3:1 (determined by ^1^H-NMR). Diagnostic ^1^H-NMR peaks of the major isomer **11**: ^1^H-NMR (300 MHz, Chloroform-*d*) δ 7.59 (dd, *J* = 4.6, 0.8 Hz, 1H), 7.26–7.22 (m, 1H), 7.18 (dd, *J* = 4.6, 3.3 Hz, 1H), 6.11 (m, 1H), 2.63–2.53 (m, 1H), 2.30–2.17 (m, 3H), 2.14 (s, 3H), 2.12–1.92 (m, 2H), 1.66–1.37 (m, 1H), 0.374 (s, 3H), 0.37 (s, 3 H); Diagnostic ^13^C-NMR peaks for the major isomer **11** (75 MHz, CDCl_3_) δ 211.4, 137.8, 136.3, 135.9, 134.7, 130.7, 128.0, 47.0, 28.3, 27.9, 26.4, 24.9, −2.4, −2.5; HRMS calcd for C_14_H_21_OSSi (M + H)^+^ 265.1082, found 265.1075.

#### 3.4.2. 5-(Dimethyl(thiophen-2-yl)silyl)-2-phenyl-3a,4,7,7a-tetrahydro-1*H*-isoindole-1,3(2*H*)-dione **15**

Diene **4** (0.116 g, 0.6 mmol) and *N*-phenylmaleimide (0.086 g, 0.5 mmol) were reacted using the general procedure. The crude product was purified using silica gel (DCM mobile phase) (R_f_ 0.37). A light yellow colored solid was obtained (0.151 g, 0.41 mmol, 82%). ^1^H-NMR (300 MHz, Chloroform-*d*) δ 7.60 (dd, *J* = 4.6, 0.9 Hz, 1H), 7.49–7.31 (m, 3H), 7.25 (dd, *J* = 3.2, 1.0 Hz, 1H), 7.20–7.08 (m, 3H), 6.38 (ddd, *J* = 6.2, 3.5, 2.5 Hz, 1H), 3.25–3.22 (m, 2H), 2.91–2.70 (m, 2H), 2.40–2.22 (m, 2H), 0.38 (s, 6H). ^13^C-NMR (75 MHz, CDCl_3_) δ 179.0, 178.6, 140.2, 138.6, 136.3, 135.0, 132.0, 131.0, 129.0, 128.4, 128.2, 126.3, 39.3, 39.2, 26.3, 25.0, −2.6, −2.7; HRMS calcd for C_14_H_21_OSSi (M + Na)^+^ 390.0960, found 390.0957.

#### 3.4.3. 6-(Dimethyl(thiophen-2-yl)silyl)-3a-methyl-2-phenyl-3a,4,7,7a-tetrahydro-1*H*-isoindole-1,3(2*H*)-dione **17** and 5-(Dimethyl(thiophen-2-yl)silyl)-3a-methyl-2-phenyl-3a,4,7,7a-tetrahydro-1*H*-isoindole-1,3(2*H*)-dione **18**

Diene **4** (0.116 g, 0.6 mmol) and 2 methyl-*N*-phenylmaleimide (0.094 g, 0.5 mmol) were reacted using the general procedure. The crude product was purified using column chromatography (silica gel, DCM, R_f_ 0.56). Compounds **17** and **18** were obtained as a light yellow solid (0.152 g, 0.4 mmol, 80%) as 2:1 mixture of isomers. Diagnostic ^1^H-NMR peaks of the major isomer **17**: ^1^H-NMR (300 MHz, Chloroform-*d*) δ 7.53 (dd, *J* = 4.6, 0.9 Hz, 1H), 7.39–7.27 (m, 3H), 7.24 (m, 1H), 7.19–6.96 (m, 3H), 6.30 (dt, *J* = 6.4, 3.1 Hz, 1H), 2.90–2.59 (m, 3H), 2.23 (m, 1H), 1.91 (m, 1H), 1.36 (s, 3H), 0.3 (s, 6H); Diagnostic ^13^C-NMR peaks of the major isomer **17**. ^13^C-NMR (75 MHz, Chloroform-*d*) δ 181.9, 177.8, 140.5, 139.2, 138.5, 135.0, 132.1, 131.0, 129.0, 128.4, 128.2, 126.3, 47.2, 44.3, 33.8, 26.8, 24.7, −2.5, −2.6; HRMS calcd for C_21_H_23_NNaO_2_SSi (M + Na)^+^ 404.1116, found 404.1112.

### 3.5. General Procedure for Cross-Coupling Diels-Alder Adducts to Produce Phenyl-Substituted Cyclohexenes *(**13**, **14**, **16**, **19**, **20**)*

In a 5 mL round bottom flask 1 equivalent of Diels-Alder adduct, 1.2 equivalents of iodobenzene, Pd(OAc)_2_ catalyst (5 mol %), and THF (3 mL) were added. A rubber septum was attached and the solution was degassed for 5 min. Two equivalents of TBAF were added and stirred at RT for 1–3 h. The completion of the reaction was monitored with thin layer chromatography. Upon completion of the reaction it was extracted with sat NaHCO_3_ soln (15 mL) and of ethyl acetate (2 × 10 mL). The organic layers were dried over Na_2_SO_4_ and concentrated under vacuum. The crude product was purified using silica gel (DCM mobile phase).

#### 3.5.1. 1-(2,3,4,5-Tetrahydro-[1,1′-biphenyl]-4-yl)ethan-1-one (**13**) and 1-(2,3,4,5-Tetrahydro-[1,1′-biphenyl]-3-yl)ethan-1-one (**14**)

The mixture of cycloadducts **11** and **12 (**0.050 g, 0.189 mmol), iodobenzene (0.046 g, 0.226 mmol), Pd(OAc)_2_ (0.002 g, 0.009 mmol), and TBAF (0.45 mL of a 1 M soln in THF, 0.45 mmol) were reacted for an hour following the general procedure. Compounds (**13** and **14**) were isolated as a colorless viscous liquid (0.034 g, 0.17 mmol, 91%), identical by ^1^H-NMR comparison to previously reported material [25].

#### 3.5.2. 2,5-Diphenyl-3a,4,7,7a-tetrahydro-1*H*-isoindole-1,3(2*H*)-dione (**16**)

Cycloadduct **15** **(**0.090 g, 0.25 mmol), iodobenzene (0.060 g, 0.294 mmol), Pd(OAc)_2_ (0.003 g, 0.013 mmol), and TBAF (0.49 mL of a 1 M soln in THF, 0.49 mmol) were reacted for 3 h following the general procedure. Compound **16** was isolated as a colorless solid (0.066 g, 0.22 mmol, 87%), identical by ^1^H-NMR comparison to previously reported material [25].

#### 3.5.3. 3a-Methyl-2,6-diphenyl-3a,4,7,7a-tetrahydro-1*H*-isoindole-1,3(2*H*)-dione (**19**) and 3a-Methyl-2,5-diphenyl-3a,4,7,7a-tetrahydro-1*H*-isoindole-1,3(2*H*)-dione (**20**)

Mixture of cycloadducts **17** and **18** (0.115 g, 0.3 mmol), iodobenzene (0.073 g, 0.36 mmol), Pd(OAc)_2_ (0.004 g, 0.018 mmol), and TBAF (0.6 mL of a 1 M soln in THF, 0.6 mmol) were reacted for 3 h following the general procedure. Compounds **19** and **20** were isolated as a colorless solid (0.084 g, 0.26 mmol, 88%), identical by ^1^H-NMR comparison to previously reported material [25].

### 3.6. Silyl Alkyne Preparation

Ethynyldimethyl(thiophen-2-yl)silane (**21**) was prepared following a published literature procedure [19].

#### Ethynyldimethyl(pyridin-2-yl)silane (**22**)

To a 250 mL flame dried flask dimethyldichlorosilane (2.6 mL, 21 mmol) was added followed by anhydrous diethyl ether (15 mL). Ethynylmagnesiumbromide (44 mL of a 0.5 M soln in THF, 22 mmol) was added and stirred at RT for 2 h. In a separate 50 mL round-bottom flask 2-bromopyridine (0.348 g, 22 mmol) was added followed by THF (5 mL). *n*-BuLi (14.5 mL of a 1.6 M soln in hexanes, 23.1 mmol) was added to the reaction mixture at −78 °C and was stirred under Ar for 10 min. The 2-lithiopyridine soln was added to the previous flask and was stirred at RT for 10 h. The resultant reaction mixture was washed with sat NaHCO_3_ (30 mL) soln. The organic layer was collected, dried over Na_2_SO_4_ and concentrated by rotary evaporation. The crude reaction mixture was distilled at 45 °C at 4 mm of Hg. Compound **22** was isolated with 90% purity (2.43 g, 15.12 mmol). Diagnostic ^1^H-NMR peaks (300 MHz, Chloroform-*d*) δ 8.54 (d, *J* = 4.9 Hz, 1H), 7.52 (m, 2H), 7.39 (t, *J* = 8.4 Hz, 1H), 2.32 (s, 1H), 0.26 (s, 6H).

### 3.7. General Procedure for Tandem Ene-Yne Cross-Metathesis and Diels-Alder Reactions

In a 5 mL flame-dried round-bottom flask, alkene (1.2 equiv), alkyne (1 equiv), and DCM (3 mL) were added and thoroughly degassed with Ar. Hoveyda-Grubbs 2nd generation catalyst (6 mol %) was added. The reaction mixture was stirred under Ar as described below. *N*-phenylmaleimide (0.9 equiv) was added and heated for 36 h. The reaction mixture was diluted with ice cold methanol and the methanol extract was filtered through a silica plug to remove catalyst and precipitated stilbene byproduct. The methanol was removed under vacuum and the crude product was purified by flash column chromatography.

*(3aR,4S,7aS)-6-(Dimethyl(thiophen-2-yl)silyl)-2,4-diphenyl-2,3,3a,4,7,7a-hexahydro-1H-isoindole* (**25a**). Ethynyldimethyl(thiophen-2-yl)silane (**21**) (0.28 g, 1.68 mmol), styrene (233 µL, 2 mmol), and catalyst (0.063 g, 0.1 mmol) were refluxed for 12 h under Ar. *N*-phenylmaleimide (0.262 g, 1.5 mmol) was added and refluxed for 40 h. After washing with cold methanol, the crude product was chromatographed on silica gel using 8:1 benzene and ethyl acetate (R_f_ 0.5). Compound **25** was obtained as a white solid (0.456 g, 1.03 mmol, 69%) along with a mixture of **25** and **26** (0.052 g) which was rechromatographed as described below. ^1^H-NMR (300 MHz, Chloroform-*d*) δ 7.63 (d, *J* = 4.6 Hz, 1H), 7.44–7.28 (m, 8H), 7.24–7.13 (m, 2H), 6.82 (m, 2H), 6.67 (dd, *J* = 4.8, 2.2 Hz, 1H), 3.85 (t, *J* = 5.9 Hz, 1H), 3.47 (dd, *J* = 9.1, 6.7 Hz, 1H), 3.37 (td, *J* = 8.7, 2.6 Hz, 1H), 3.07 (dd, *J* = 16.5, 2.6 Hz, 1H), 2.51 (ddt, *J* = 16.4, 8.4, 2.1 Hz, 1H), 0.47 (s, 6H). ^13^C-NMR (75 MHz, CDCl_3_) δ 178.1, 176.1, 140.4, 139.6, 138.6, 136.2, 135.2, 131.7, 131.2, 129.1, 128.8, 128.8, 128.4, 128.3, 127.2, 126.2, 45.2, 42.2, 39.0, 25.6, −2.4, −2.6; HRMS calcd for C_26_H_26_NO_2_SSi (M + H)^+^ 444.1454, found 444.1449.

*(3aS,4S,7aR)-6-(Dimethyl(thiophen-2-yl)silyl)-2,4-diphenyl-2,3,3a,4,7,7a-hexahydro-1H-isoindole* (**26a**). After isolating **25** as described above, the remaining column fractions (0.052 g) were chromatographed using 4:1 pentane ethyl acetate to yield additional **25** (0.034 g, 0.08 mmol, 5%) (R_f_ 0.5) followed by compound **26** (R_f_ 0.42) isolated as white solid (0.018 g, 0.04 mmol, 3%). ^1^H-NMR (500 MHz, Chloroform-*d*) δ 7.63 (d, *J* = 4.8, 1H), 7.45 (t, *J* = 7.6 Hz, 2H), 7.40–7.34 (m, 3H), 7.33–7.27 (m, 4H), 7.22–7.18 (m, 3H), 6.55–6.50 (m, 1H), 4.12 (m, 1H), 3.44 (dd, *J* = 9.3, 3.6 Hz, 1H), 3.19 (ddd, *J* = 9.3, 7.6, 4.8 Hz, 1H), 2.64–2.47 (m, 2H), 0.45 (s, 6H). ^13^C-NMR (126 MHz, CDCl_3_) δ 178.5, 178.1, 141.2, 141.0, 139.8, 136.2, 135.1, 131.9, 131.2, 129.0, 128.8, 128.5, 128.35, 127.6, 126.8, 126.3, 46.6, 41.3, 39.0, 26.2, −2.6, −2.7; HRMS calcd for C_26_H_26_NO_2_SSi (M + H)^+^ 444.1454, found 444.1449.

*(3aR,4S,7aS)-6-(Dimethyl(thiophen-2-yl)silyl)-4-(4-methoxyphenyl)-2-phenyl-2,3,3a,4,7,7a-hexahydro-1H-isoindole* (**25b**). Ethynyldimethyl(thiophen-2-yl)silane (**21**) (0.28 g, 1.68 mmol), *p*-vinyl-anisole (263 µL, 2 mmol), and catalyst (0.063 g, 0.1 mmol) were refluxed under Ar for 3 h. *N*-phenylmaleimide (0.262 g, 1.5 mmol) was added and refluxed for 40 h. The crude product was chromatographed on silica gel using 8:1 benzene:ethyl acetate (R_f_ 0.48). Compound **25b** was isolated as a light yellow solid (0.506 g, 1.07 mmol, 71%). ^1^H-NMR (300 MHz, Chloroform-*d*) δ 7.64 (dd, *J* = 4.6, 0.9 Hz, 1H), 7.39–7.28 (m, 4H), 7.21 (dd, *J* = 4.6, 3.3 Hz, 1H), 7.18–7.11 (m, 2H), 6.84 (m, 4H), 6.63 (dd, *J* = 4.9, 2.1 Hz, 1H), 3.86–3.79 (m, 1H), 3.78 (s, 3H), 3.50–3.25 (m, 2H), 3.06 (dd, *J* = 16.5, 2.6 Hz, 1H), 2.52 (ddt, *J* = 16.5, 8.2, 1.8 Hz, 1H), 0.47 (s, 6H). ^13^C-NMR (126 MHz, CDCl_3_) δ 178.3, 176.3, 158.8, 140.8, 139.2, 135.2, 131.7, 131.2, 130.4, 130.1, 128.9, 128.3, 127.4, 126.3, 113.8, 55.2, 45.3, 41.5, 39.0, 25.5, −2.4, −2.5; HRMS calcd for C_27_H_28_NO_3_SSi (M + H)^+^ 474.1559, found 474.1557.

*(3aR,4S,7aS)-4-(4-Chlorophenyl)-6-(dimethyl(thiophen-2-yl)silyl)-2-phenyl-3a,4,7,7a-tetrahydro-1H-isoindole-1,3(2H)-dione* (**25c**). Ethynyldimethyl(thiophen-2-yl)silane (**21**) (0.07 g, 0.42 mmol), *p*-chloro-styrene (60 µL, 0.5 mmol), and catalyst (0.016 g, 0.025 mmol) were refluxed under Ar for 30 h. *N*-phenylmaleimide (0.066 g, 0.38 mmol) was added and refluxed for 40 h. The crude product was chromatographed on silica gel using 8:1 benzene:ethyl acetate (R_f_ 0.52) and isolated as a white solid (0.112 g, 0.23 mmol, 61%). ^1^H-NMR (300 MHz, Chloroform-*d*) δ 7.64 (dd, *J* = 4.6, 0.9 Hz, 1H), 7.31 (m, 7H), 7.24–7.18 (m, 2H), 6.82 (m, 2H), 6.67 (dd, *J* = 4.7, 2.1 Hz, 1H), 3.86 (t, *J* = 5.7 Hz, 1H), 3.48 (dd, *J* = 9.1, 6.7 Hz, 1H), 3.38 (td, *J* = 8.8, 2.6 Hz, 1H), 3.08 (dd, *J* = 16.5, 2.6 Hz, 1H), 2.52 (dd, *J* = 16.5, 8.5 Hz, 1H), 0.48 (s, 6H). ^13^C-NMR (75 MHz, CDCl_3_) δ 178.2, 176.1, 140.4, 139.5, 138.5, 136.2, 135.2, 131.6, 131.2, 129.1, 128.9, 128.4, 128.37, 128.34, 127.2, 126.0, 45.2, 42.2, 39.0, 25.6, −2.4, −2.6; HRMS calcd for C_26_H_24_ClNNaO_2_SSi (M + Na)^+^ 500.0883, found 500.0901.

### 3.8. General Procedure for Cross-Coupling of ***25a**–**c***

In a 5 mL round-bottom flask, 1 equivalent of Diels-Alder adduct, 1.2 equivalent of iodobenzene, Pd(OAc)_2_ catalyst (5 mol %), and THF (3 mL) were added. A rubber septum was attached and it was degassed for 5 min. Two equivalents of TBAF were added and stirred for at RT for 15 min. The septum was removed and a reflux condenser was attached. The reaction mixture was refluxed for 4 h. Upon completion of the reaction it was washed with sat NaHCO_3_ soln (15 mL) and extracted with ethyl acetate (10 mL). The organic layers were dried over Na_2_SO_4_ and concentrated via rotary evaporator. The crude product was purified using silica gel with a pentane/ethyl acetate mobile phase.

#### 3.8.1. (3a*R*,4*R*,7a*S*)-2,4,6-Triphenyl-3a,4,7,7a-tetrahydro-1*H*-isoindole-1,3(2*H*)-dione (**27a**)

Cycloadduct **25a** (0.1 g, 0.23 mmol), iodobenzene (0.057 g, 0.28 mmol), Pd(OAc)_2_ (0.003 g, 0.013 mmol), and TBAF (0.46 mL of a 1 M soln in THF, 0.46 mmol) were reacted following the general procedure. Compound **27a** was isolated as white colored solid (0.063 g, 0.17 mmol, 72%), identical by ^1^H-NMR comparison to previously reported material [11]. 

#### 3.8.2. (3a*R*,4*R*,7a*S*)-4-(4-Methoxyphenyl)-2,6-diphenyl-3a,4,7,7a-tetrahydro-1*H*-isoindole-1,3(2*H*)-dione (**27b**)

Cycloadduct **25b** **(**0.095 g, 0.2 mmol), iodobenzene (0.049 g, 0.24 mmol), Pd(OAc)_2_ (0.002 g, 0.01 mmol), and TBAF (0.40 mL of a 1 M soln in THF, 0.4 mmol) were reacted following the general procedure. Compound **27b** was isolated as a white solid (0.064 g, 0.16 mmol, 78%), identical by ^1^H-NMR comparison to previously reported material [11]. 

#### 3.8.3. (3a*R*,4*R*,7a*S*)-4-(4-Chlorophenyl)-2,6-diphenyl-3a,4,7,7a-tetrahydro-1*H*-isoindole-1,3(2*H*)-dione (**27c**)

Cycloadduct **25c** **(**0.106 g, 0.22 mmol), iodobenzene (0.054 g, 0.26 mmol), Pd(OAc)_2_ (0.003 g, 0.013 mmol), and TBAF (0.44 mL of a 1 M soln in THF, 0.44 mmol) were reacted following the general procedure. Compound **27c** was isolated as a white solid (0.057 g, 0.14 mmol, 65%), identical by ^1^H-NMR comparison to previously reported material [11].

## 4. Conclusions

2-Silicon-substituted 1,3-dienes containing nontransferrable groups known to promote transmetallation were prepared by Grignard chemistry and enyne metathesis. Dienes produced participated in Diels-Alder reactions in highly regio- and diastereoselective fashions. Electron-rich alkenes showed the fastest rates in enyne metathesis reactions, and ethylene, a commonly used promoter, slowed enyne metathesis. 2-Pyridyldimethylsilyl and 2-thienyldimethylsilyl-substituted Diels-Alder cycloadducts participated in cross-coupling chemistry and the 2-thienyldimethylsilyl-substituted cycloadducts underwent cross-coupling at room temperature, the first such cycloadducts prepared in our laboratories to do so.

## Supplementary Materials

Supplementary materials can be accessed at: http://www.mdpi.com/1420-3049/20/09/16892/s1.

## References

[1] Welker M.E. (1997). Preparation and exo-selective [4 + 2] cycloaddition reactions of cobaloxime-substituted 1,3-dienes. Adv. Cycloaddit..

[2] Welker M.E. (2001). Organocobalt complexes in organic synthesis. Curr. Org. Chem..

[3] Welker M.E. (2008). Recent advances in syntheses and reaction chemistry of boron and silicon substituted 1,3-dienes. Tetrahedron.

[4] Lim D.S.W., Anderson E.A. (2012). Synthesis of vinylsilanes. Synth. Stuttg..

[5] Herndon J.W. (2012). The chemistry of the carbon-transition metal double and triple bond: Annual survey covering the year 2010. Coord. Chem. Rev..

[6] Sore H.F., Galloway W., Spring D.R. (2012). Palladium-catalysed cross-coupling of organosilicon reagents. Chem. Soc. Rev..

[7] Puri J.K., Singh R., Chahal V.K. (2011). Silatranes: A review on their synthesis, structure, reactivity and applications. Chem. Soc. Rev..

[8] Pidaparthi R.R., Welker M.E. (2007). Preparation of siloxacyclopentene containing 1,3-dienes and their Diels-Alder reactions. Tetrahedron Lett..

[9] Pidaparthi R.R., Welker M.E., Day C.S., Wright M.W. (2007). Preparation of 2-trialkylsiloxy-substituted 1,3-dienes and their Diels-Alder/cross-coupling reactions. Org. Lett..

[10] Pidaparthi R.R., Junker C.S., Welker M.E., Day C.S., Wright M.W. (2009). Preparation of 2-silicon-substituted 1,3-dienes and their Diels-Alder/cross-coupling reactions. J. Org. Chem..

[11] Junker C.S., Welker M.E., Day C.S. (2010). Synthesis of 4-aryl- and 4-alkyl-2-silyl-1,3-butadienes and their Diels-Alder/cross-coupling reactions. J. Org. Chem..

[12] Junker C.S., Welker M.E. (2012). Ruthenium carbenes as catalysts in stereoselective ene-yne metathesis/Diels-Alder and ene-yne metathesis/Diels-Alder/cross coupling multicomponent reactions. Tetrahedron.

[13] Choudhury P.P., Junker C.S., Pidaparthi R.R., Welker M.E. (2014). Syntheses of 2-silicon-substituted 1,3-dienes. J. Organomet. Chem..

[14] Hanson J.R. (2003). Diterpenoids. Nat. Prod. Rep..

[15] Denmark S.E., Choi J.Y. (1999). Highly stereospecific, cross-coupling reactions of alkenylsilacyclobutanes. J. Am. Chem. Soc..

[16] Itami K., Mitsudo K., Kamei T., Koike T., Nokami T., Yoshida J. (2000). Highly efficient carbopalladation across vinylsilane: Dual role of the 2-pyme2si group as a directing group and as a phase tag. J. Am. Chem. Soc..

[17] Hosoi K., Nozaki K., Hiyama T. (2002). Alkenyldimethyl(2-thienyl)silanes, excellent coupling partner for the palladium-catalyzed cross-coupling reaction. Chem. Lett..

[18] Ostrowska S., Powała B., Jankowska-Wajda M., Żak P., Rogalski S., Wyrzykiewicz B., Pietraszuk C. (2015). Regio- and steroselective cross-metathesis of silylacetylenes with terminal olefins and alpha, omega-dienes. J. Organomet. Chem..

[19] Denmark S.E., Tymonko S.A. (2005). Sequential cross-coupling of 1,4-bissilylbutadienes: Synthesis of unsymmetrical 1,4-disubstituted 1,3-butadienes. J. Am. Chem. Soc..

[20] Clark J.R., Diver S.T. (2011). Atom economy in the metathesis cross-coupling of alkenes and alkynes. Org. Lett..

[21] Giessert A.J., Diver S.T. (2005). Cross enyne metathesis of para-substituted styrenes: A kinetic study of enyne metathesis. Org. Lett..

[22] Mori M., Sakakibara N., Kinoshita A. (1998). Remarkable effect of ethylene gas in the intramolecular enyne metathesis of terminal alkynes. J. Org. Chem..

[23] Kinoshita A., Sakakibara N., Mori M. (1997). Novel 1,3-Diene Synthesis from alkyne and ethylene by ruthenium-catalyzed enyne metathesis. J. Am. Chem. Soc..

[24] Gregg T.M., Keister J.B., Diver S.T. (2013). Inhibitory effect of ethylene in ene-yne metathesis: The case for ruthenacyclobutane resting states. J. Am. Chem. Soc..

[25] De S., Welker M.E. (2005). Preparation of 2-BF3-substituted 1,3-dienes and their Diels−Alder/cross-coupling reactions. Org. Lett..

