# Supplementary material for: Preparation and Reaction Chemistry of Novel Silicon-Substituted 1,3-Dienes
Source: Molecules. 2015 Sep 16;20(9):16892–907. doi: 10.3390/molecules200916892 (PMC6332380; doi:10.3390/molecules200916892)
Supplement: Supplementary file 1 [file molecules-20-16892-s001.pdf]

Preparation and Reaction Chemistry of Novel Silicon Substituted 1,3-Dienes

Partha P. Choudhury and Mark E. Welker*

Department of Chemistry, Wake Forest University, P.O. Box 7486, Winston-Salem, NC 27109 (USA), email: welker@wfu.edu, FAX: (336)-758-4656

Supplementary Data

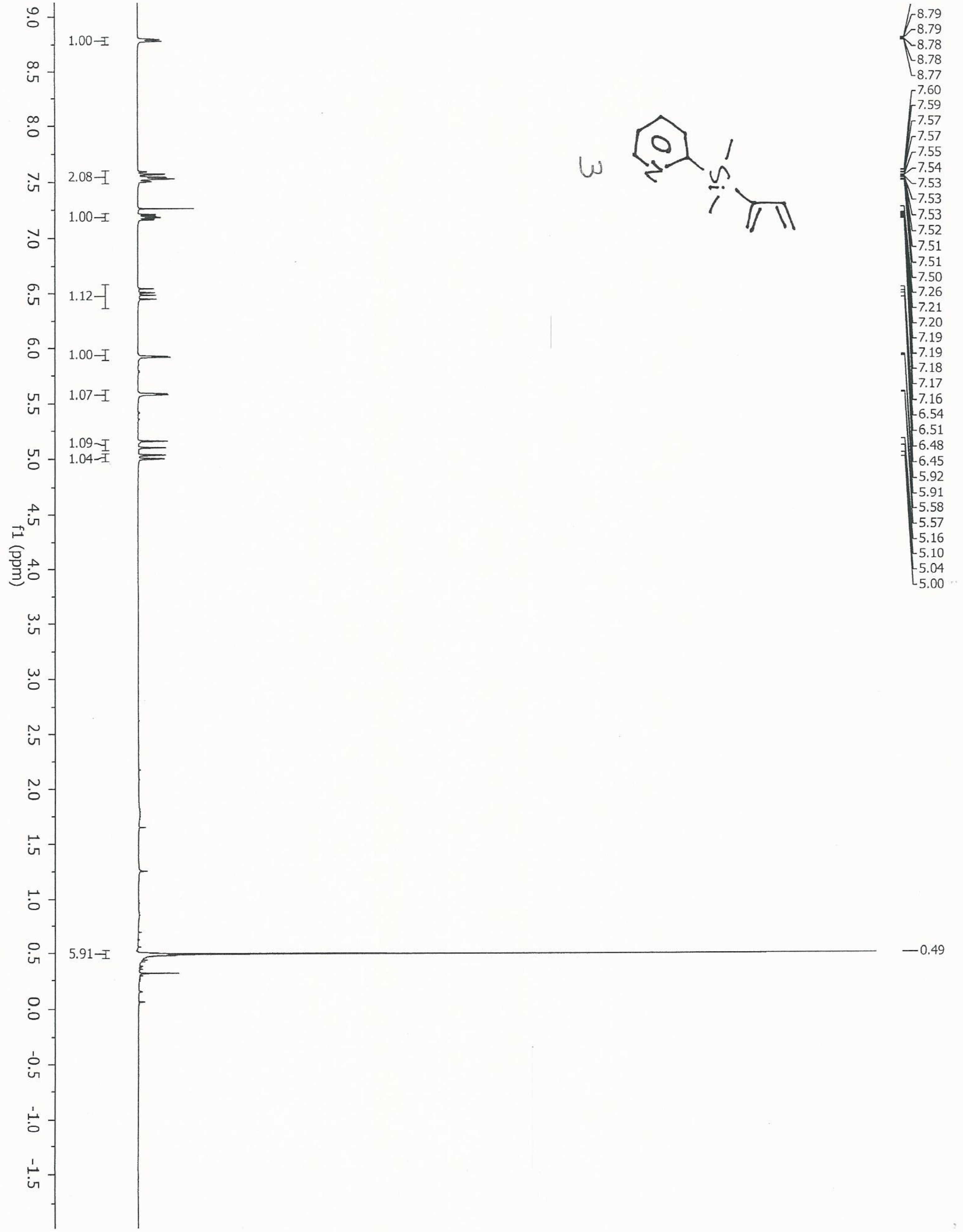

3
Si
8.79
8.79
8.78
8.78
8.77
7.60
7.59
7.57
7.57
7.55
7.54
7.53
7.53
7.53
7.52
7.51
7.51
7.50
7.26
7.21
7.20
7.19
7.19
7.18
7.17
7.16
6.54
6.51
6.48
6.45
5.92
5.91
5.58
5.57
5.16
5.10
5.04
5.00
0.49
1.00
2.08
1.00
1.12
1.00
1.07
1.09
1.04
5.91
f1 (ppm)

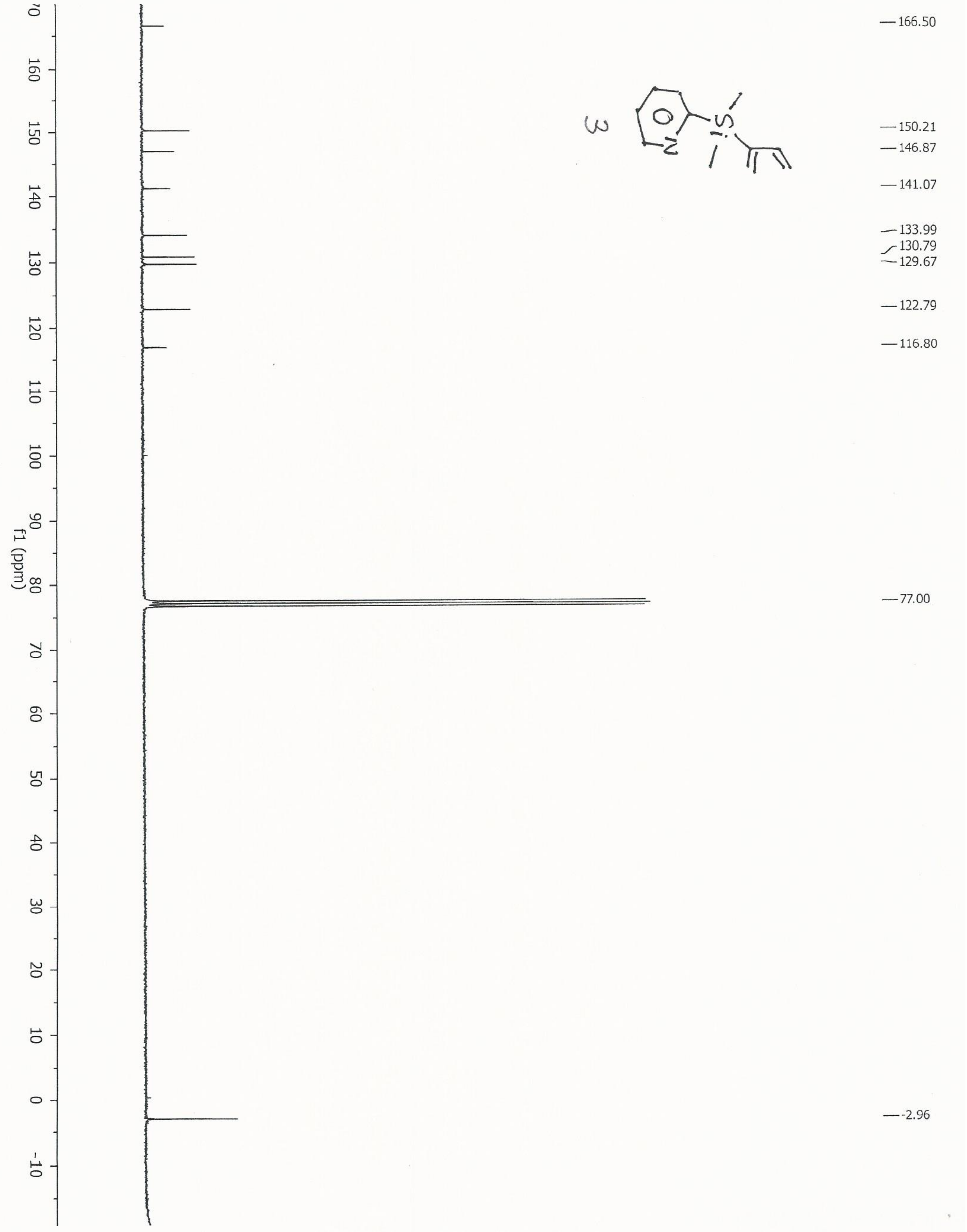

166.50
150.21
146.87
141.07
133.99
130.79
129.67
122.79
116.80
77.00
-2.96
3
Si
N
f1 (ppm)

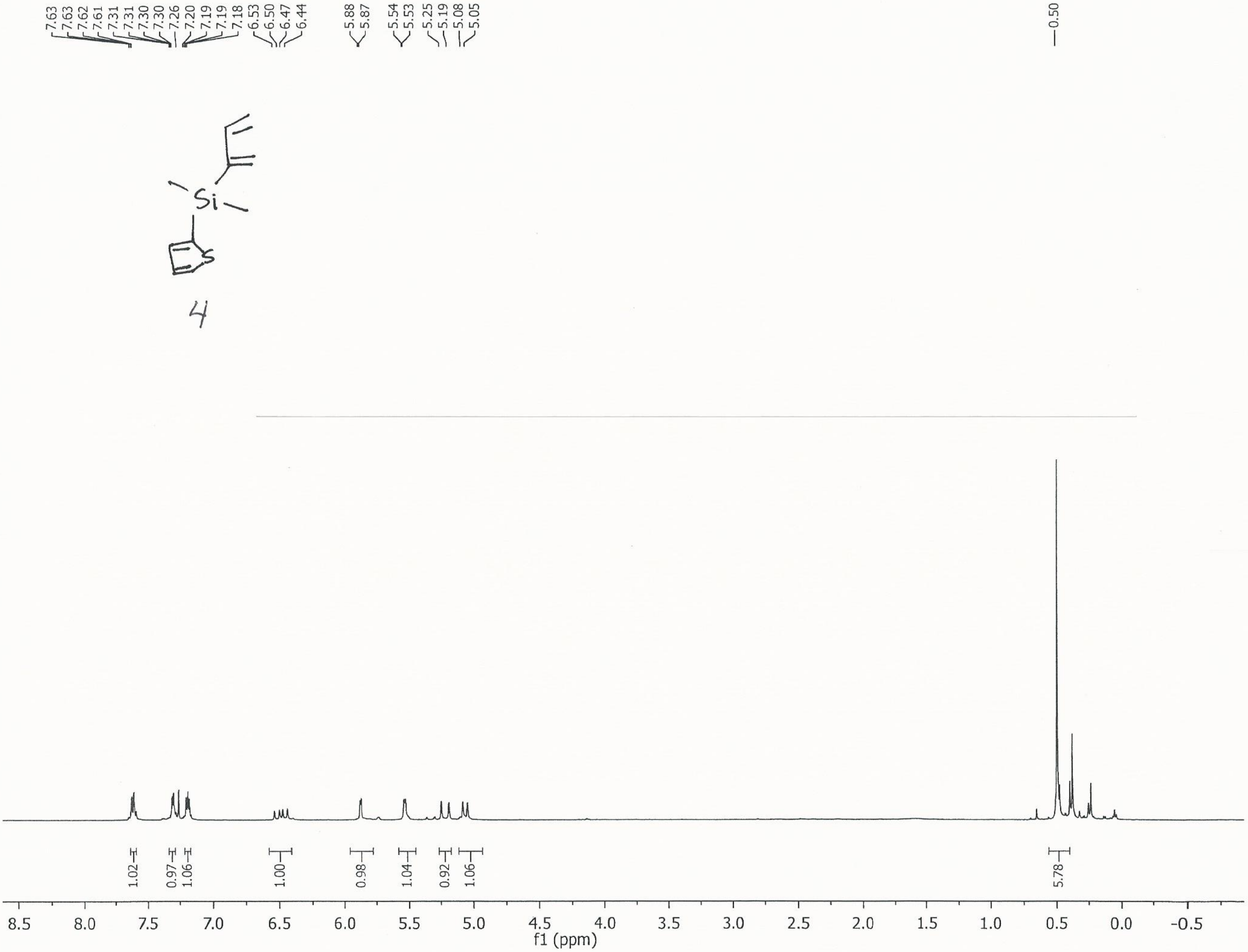
7.63
7.63
7.62
7.61
7.31
7.31
7.30
7.30
7.26
7.20
7.19
7.19
7.18
6.53
6.50
6.47
6.44
5.88
5.87
5.54
5.53
5.25
5.19
5.08
5.05
0.50
Si
S
4
1.02
0.97
1.06
1.00
0.98
1.04
0.92
1.06
5.78
8.5
8.0
7.5
7.0
6.5
6.0
5.5
5.0
4.5
4.0
3.5
3.0
2.5
2.0
1.5
1.0
0.5
0.0
-0.5
f1 (ppm)

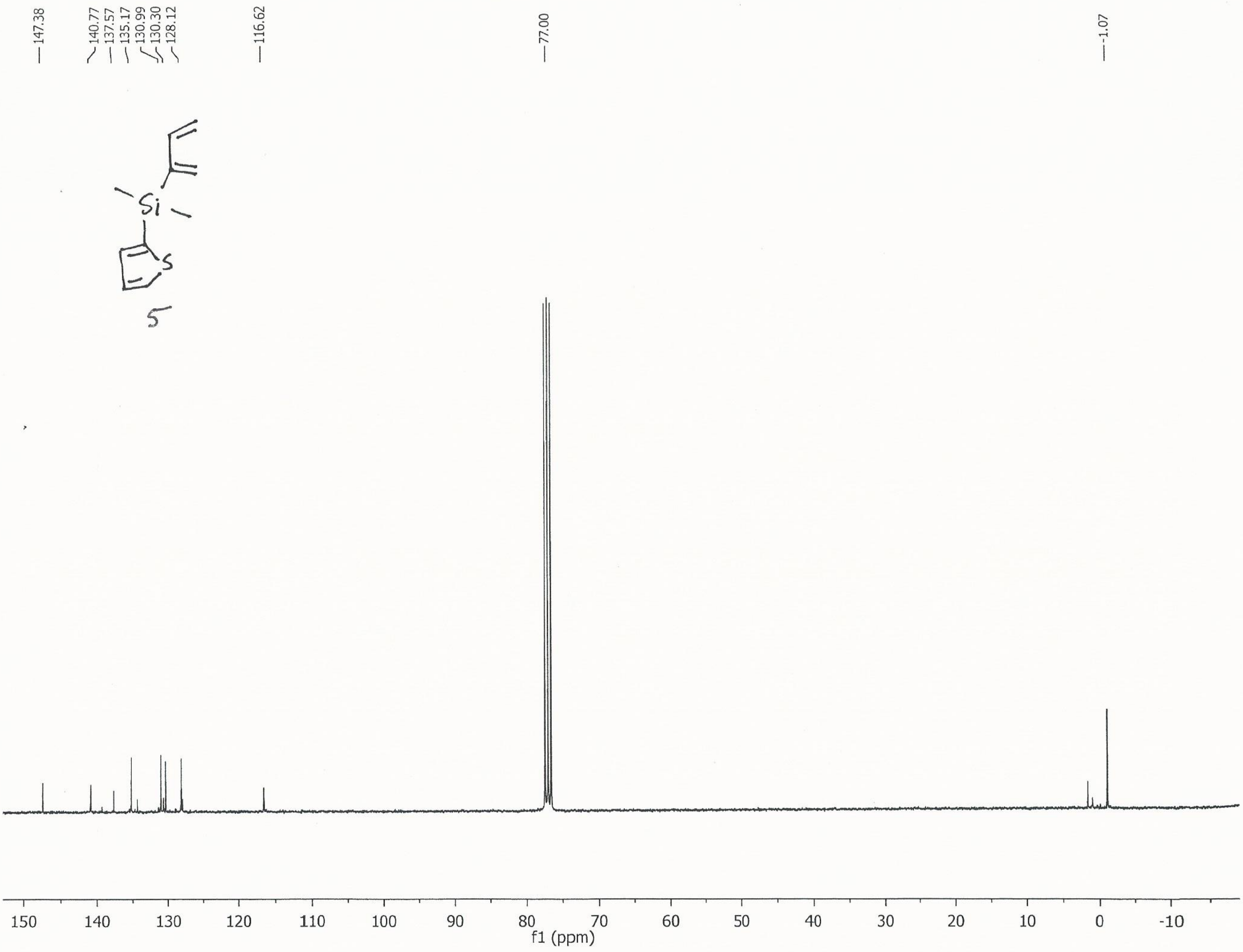
147.38
140.77
137.57
135.17
130.99
130.30
128.12
116.62
77.00
-1.07
Si
S
5
150
140
130
120
110
100
90
80
70
60
50
40
30
20
10
0
-10
f1 (ppm)

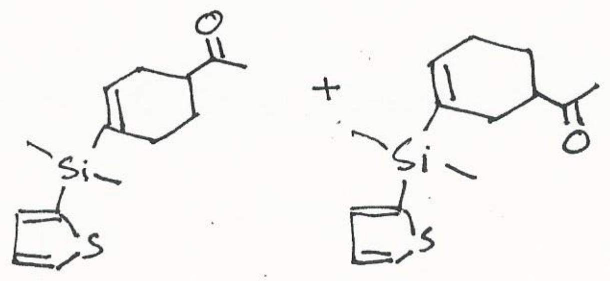

11 + 12

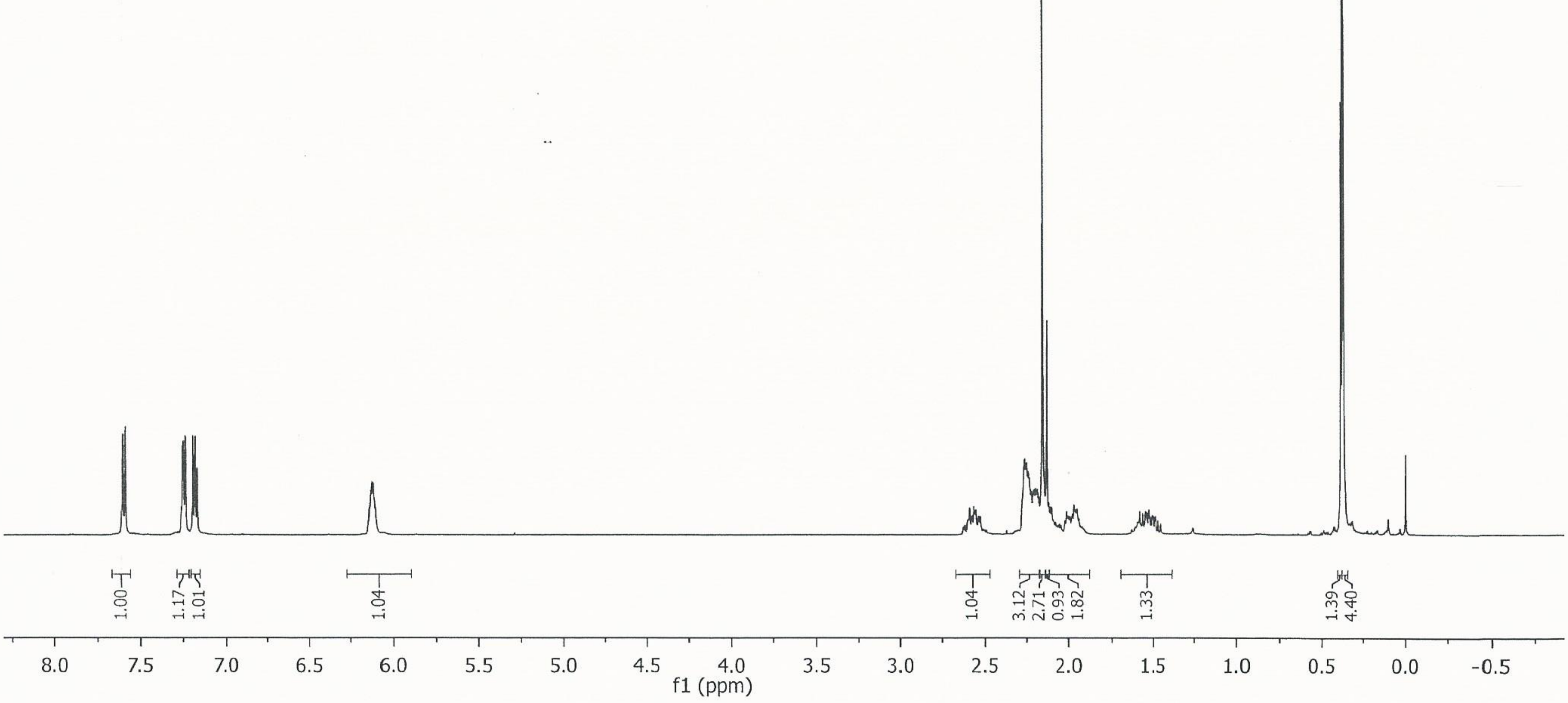
7.60
7.60
7.59
7.59
7.26
7.25
7.25
7.24
7.24
7.19
7.18
7.18
7.17
6.12
6.10
2.63
2.60
2.58
2.55
2.54
2.53
2.27
2.26
2.24
2.22
2.21
2.20
2.19
2.19
2.18
2.16
2.11
2.06
2.00
1.94
1.60
1.58
1.54
1.53
1.48
1.42
0.37
0.37
-0.00
1.00
1.17
1.01
1.04
1.04
3.12
2.71
0.93
1.82
1.33
1.39
4.40
8.0
7.5
7.0
6.5
6.0
5.5
5.0
4.5
4.0
3.5
3.0
2.5
2.0
1.5
1.0
0.5
0.0
-0.5
f1 (ppm)

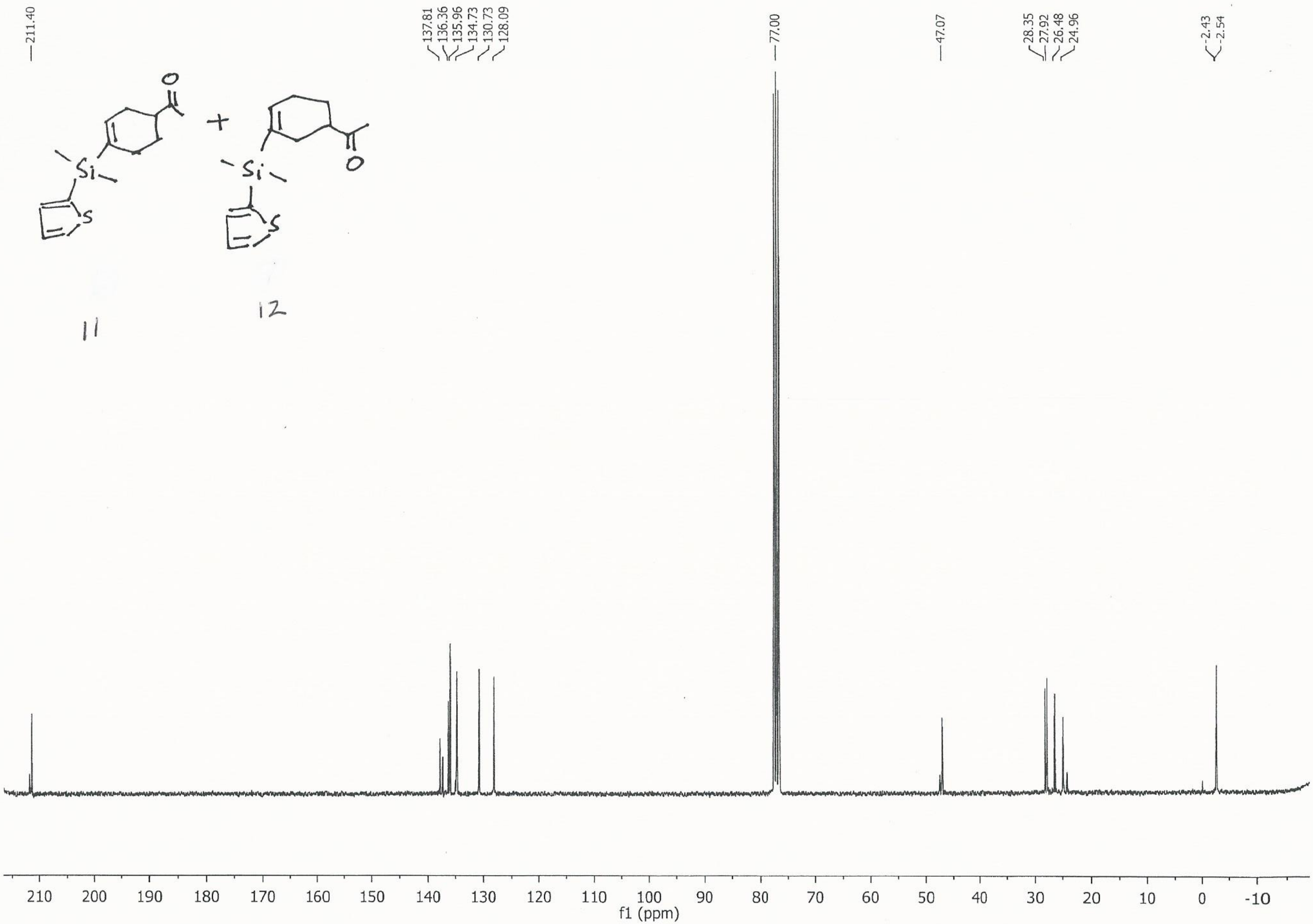
211.40
137.81
136.36
135.96
134.73
130.73
128.09
77.00
47.07
28.35
27.92
26.48
24.96
-2.43
-2.54
Si
S
O
+
11
12
210 200 190 180 170 160 150 140 130 120 110 100 90 80 70 60 50 40 30 20 10 0 -10
f1 (ppm)

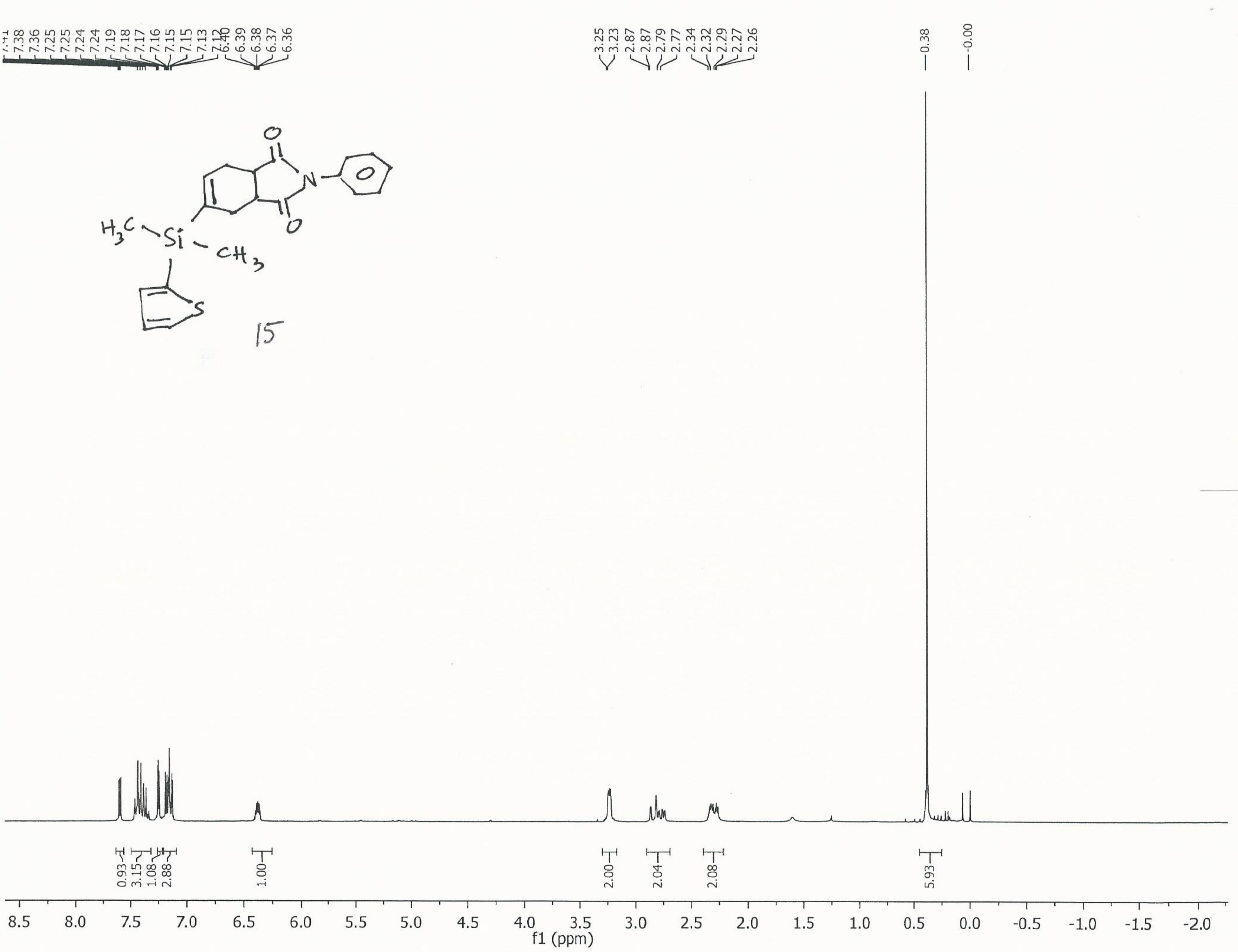

7.41
7.38
7.36
7.25
7.25
7.24
7.24
7.19
7.18
7.17
7.16
7.15
7.15
7.13
7.12
6.40
6.39
6.38
6.37
6.36
3.25
3.23
2.87
2.87
2.79
2.77
2.34
2.32
2.29
2.27
2.26
0.38
-0.00
0.93
3.15
1.08
2.88
1.00
2.00
2.04
2.08
5.93
8.5
8.0
7.5
7.0
6.5
6.0
5.5
5.0
4.5
4.0
3.5
3.0
2.5
2.0
1.5
1.0
0.5
0.0
-0.5
-1.0
-1.5
-2.0
f1 (ppm)
H_3C
Si
CH_3
N
O
O
S

15

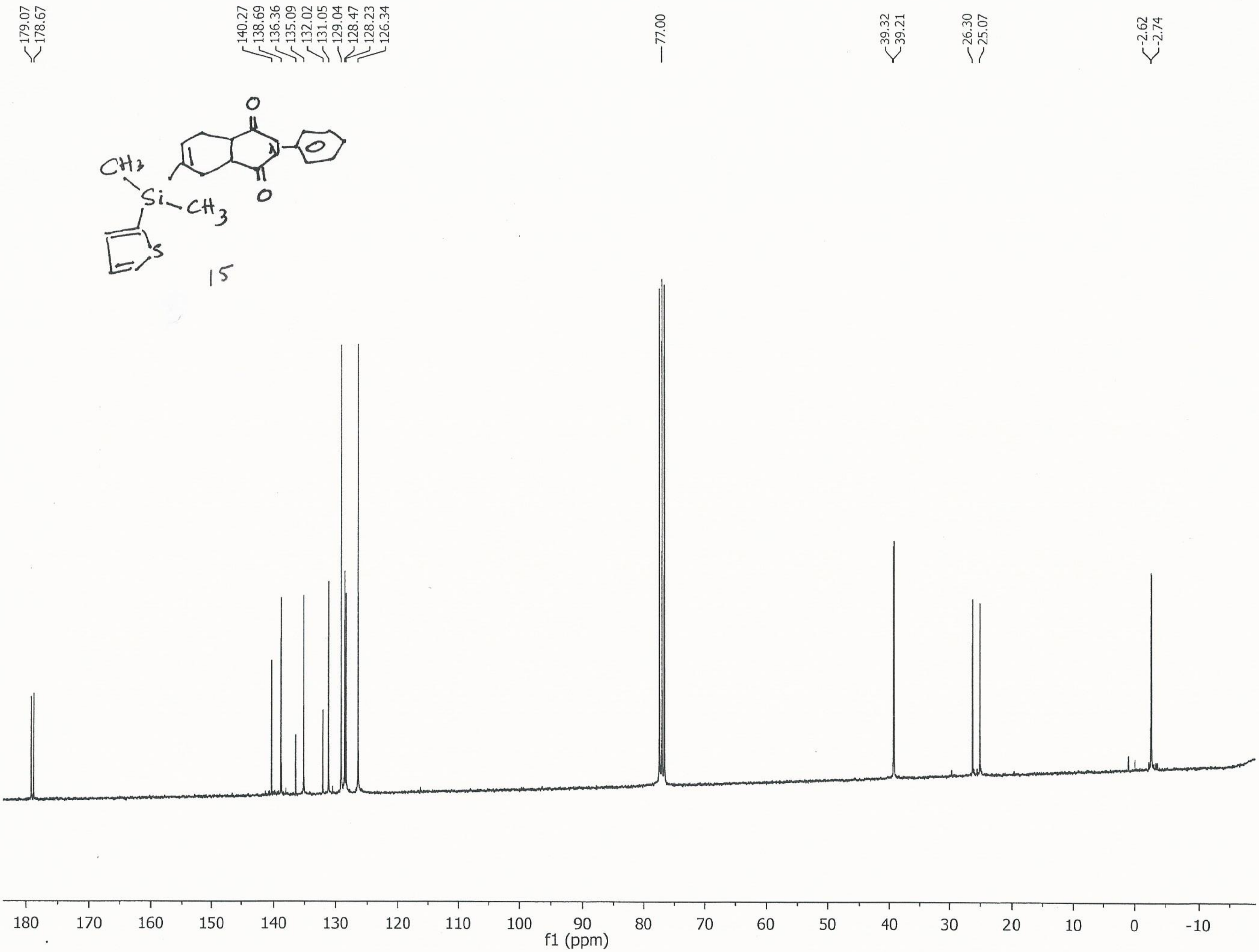
179.07
178.67
140.27
138.69
136.36
135.09
132.02
131.05
129.04
128.47
128.23
126.34
77.00
39.32
39.21
26.30
25.07
-2.62
-2.74
CH3
Si
CH3
S
O
O
15
180
170
160
150
140
130
120
110
100
90
80
70
60
50
40
30
20
10
0
-10
f1 (ppm)

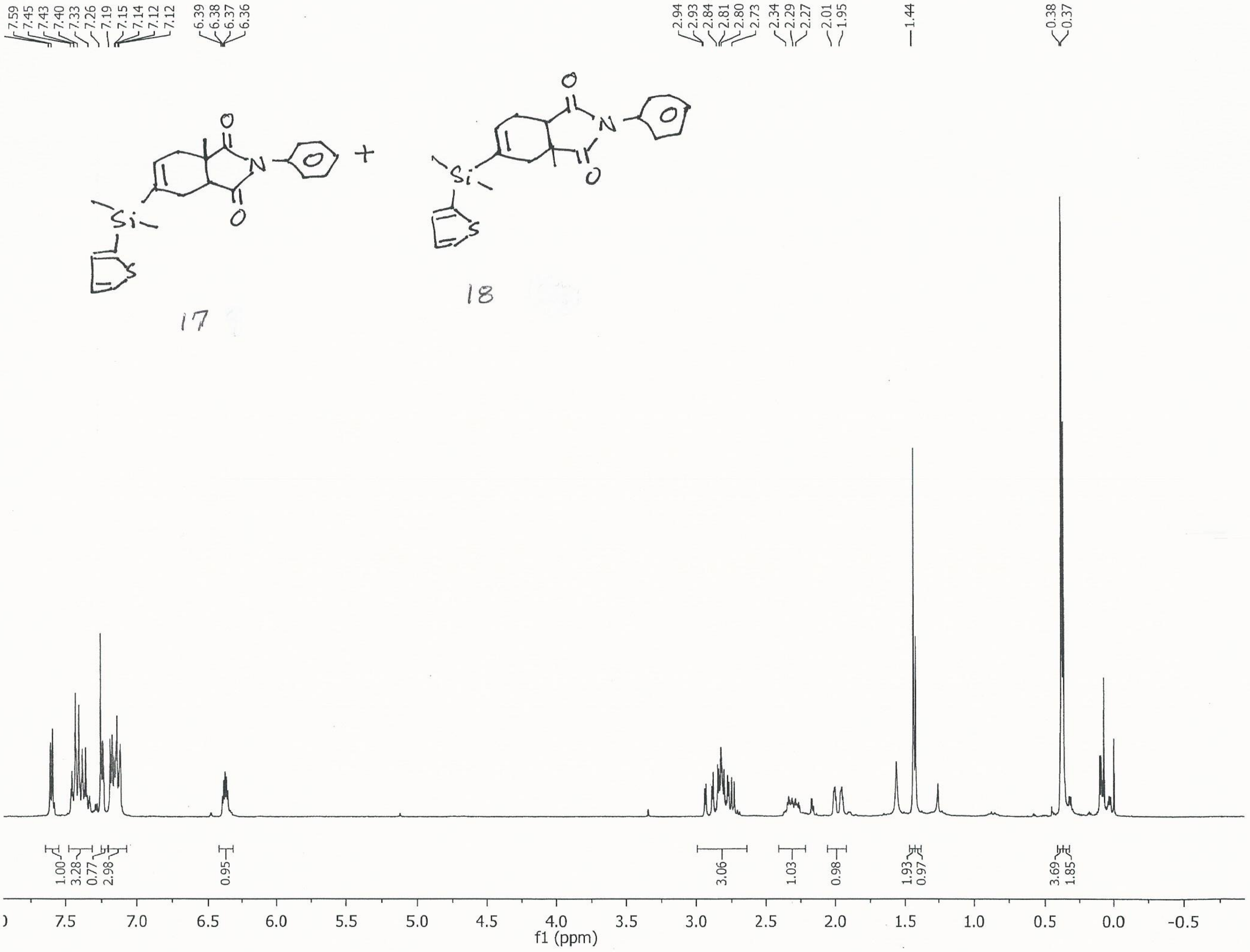

7.59
7.45
7.43
7.40
7.33
7.26
7.19
7.15
7.14
7.12
7.12
6.39
6.38
6.37
6.36
2.94
2.93
2.84
2.81
2.80
2.73
2.34
2.29
2.27
2.01
1.95
1.44
0.38
0.37
17
18
1.00
3.28
0.77
2.98
0.95
3.06
1.03
0.98
1.93
0.97
3.69
1.85
7.5
7.0
6.5
6.0
5.5
5.0
4.5
4.0
3.5
3.0
2.5
2.0
1.5
1.0
0.5
0.0
-0.5
f1 (ppm)

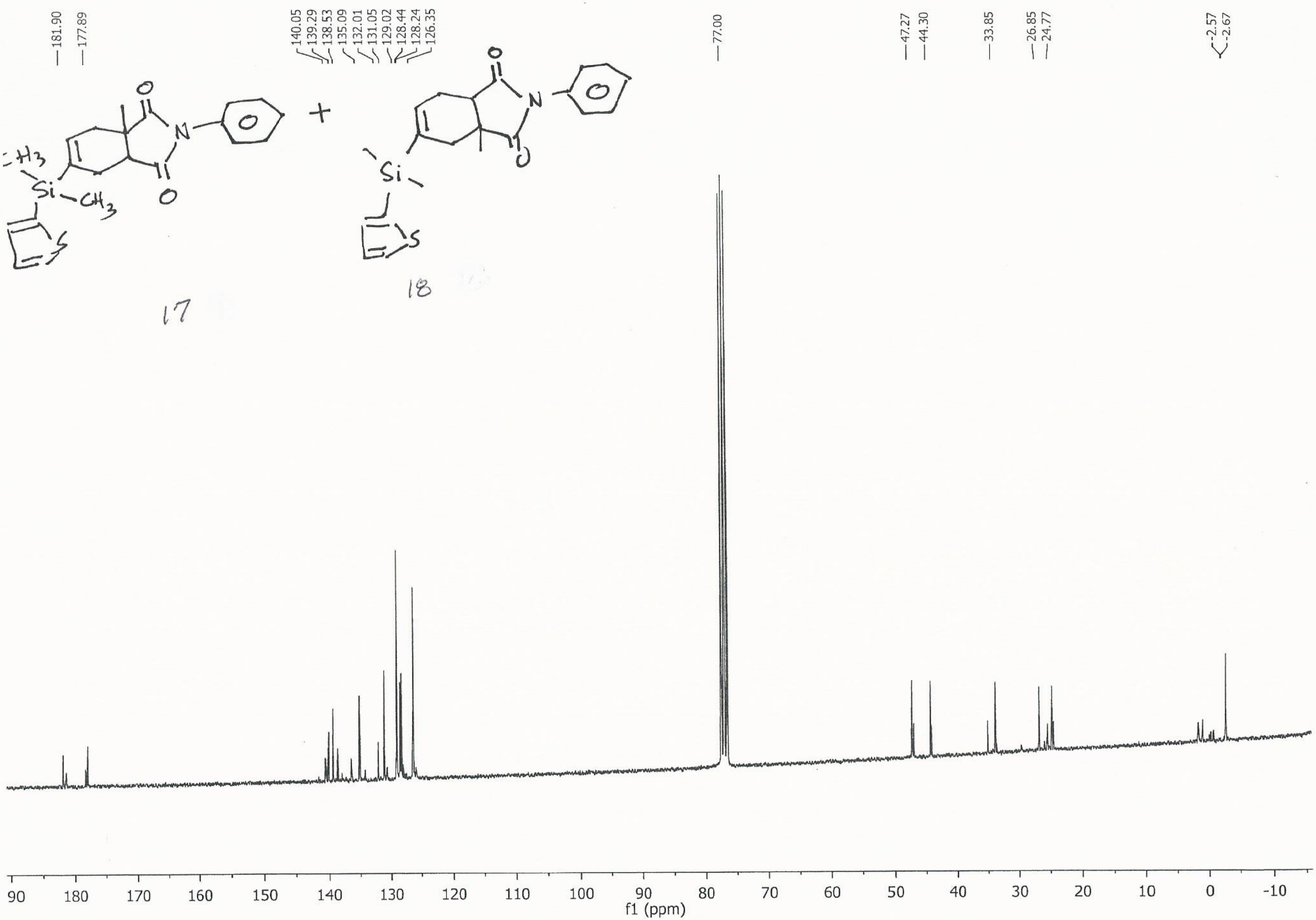
181.90
177.89
140.05
139.29
138.53
135.09
132.01
131.05
129.02
128.44
128.24
126.35
77.00
47.27
44.30
33.85
26.85
24.77
-2.57
-2.67
17
18
90 180 170 160 150 140 130 120 110 100 90 80 70 60 50 40 30 20 10 0 -10
f1 (ppm)

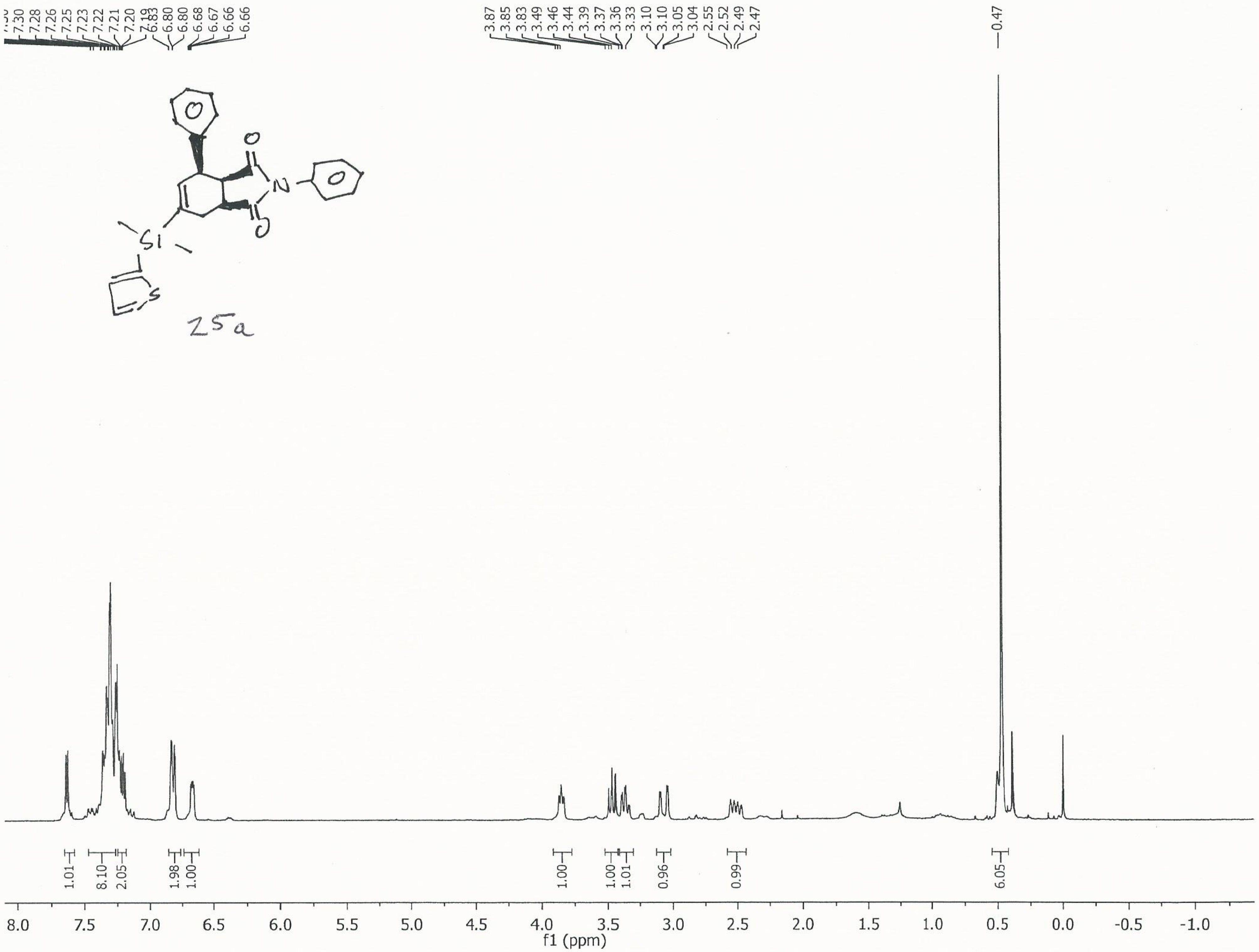

7.30
7.28
7.26
7.25
7.23
7.22
7.21
7.20
7.19
6.83
6.80
6.80
6.68
6.67
6.66
6.66
3.87
3.85
3.83
3.49
3.46
3.44
3.39
3.37
3.36
3.33
3.10
3.10
3.05
3.04
2.55
2.52
2.49
2.47
0.47
25a
1.01
8.10
2.05
1.98
1.00
1.00
1.00
1.01
0.96
0.99
6.05
8.0
7.5
7.0
6.5
6.0
5.5
5.0
4.5
4.0
3.5
3.0
2.5
2.0
1.5
1.0
0.5
0.0
-0.5
-1.0
f1 (ppm)

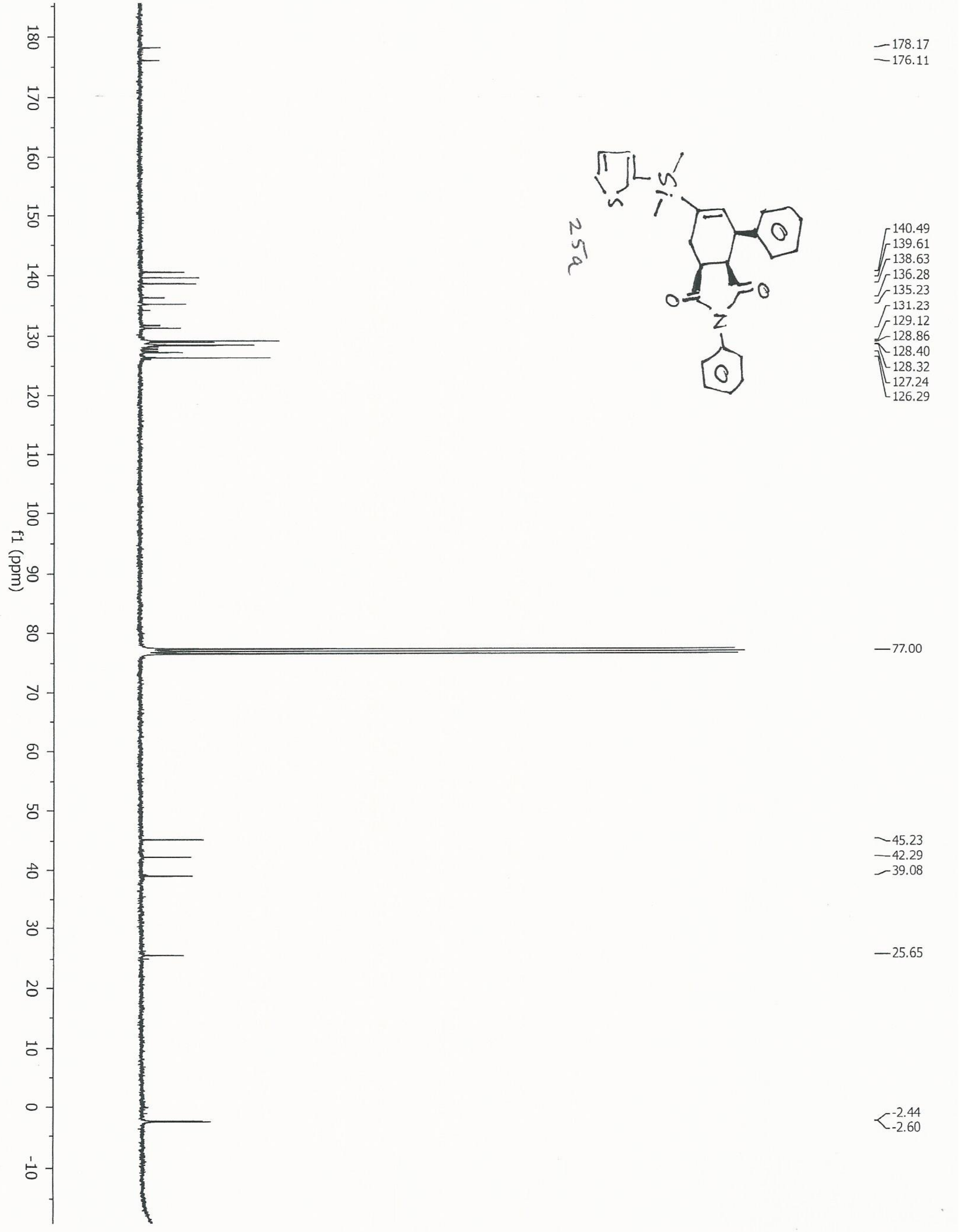
178.17
176.11
140.49
139.61
138.63
136.28
135.23
131.23
129.12
128.86
128.40
128.32
127.24
126.29
77.00
45.23
42.29
39.08
25.65
-2.44
-2.60
f1 (ppm)
25a

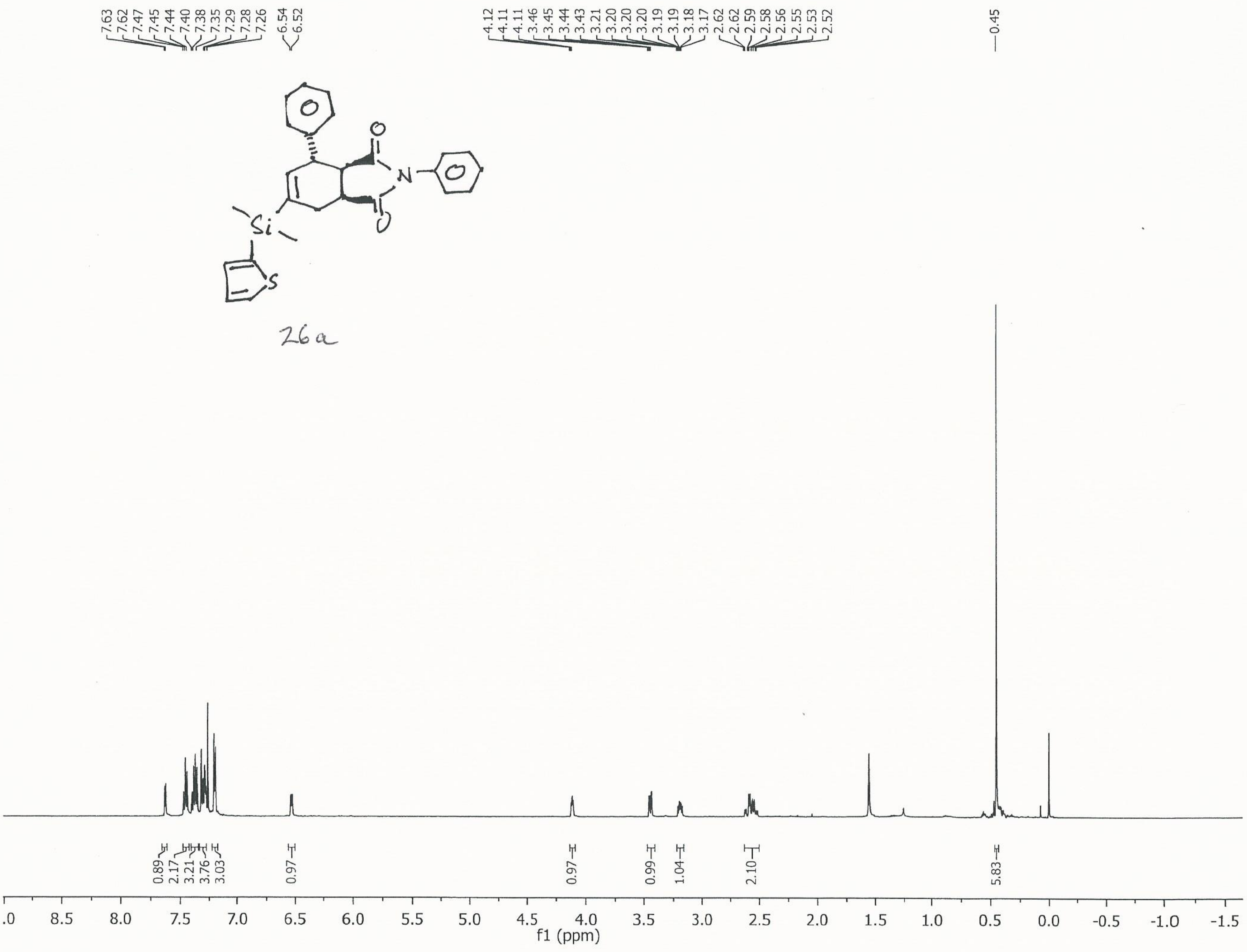

7.63
7.62
7.47
7.45
7.44
7.40
7.38
7.35
7.29
7.28
7.26
6.54
6.52
4.12
4.11
4.11
3.46
3.45
3.44
3.43
3.21
3.20
3.20
3.20
3.19
3.19
3.18
3.17
2.62
2.62
2.59
2.58
2.56
2.55
2.53
2.52
0.45
N
Si
S
26a
0.89
2.17
3.21
3.76
3.03
0.97
0.97
0.99
1.04
2.10
5.83
.0
8.5
8.0
7.5
7.0
6.5
6.0
5.5
5.0
4.5
4.0
3.5
3.0
2.5
2.0
1.5
1.0
0.5
0.0
-0.5
-1.0
-1.5
f1 (ppm)

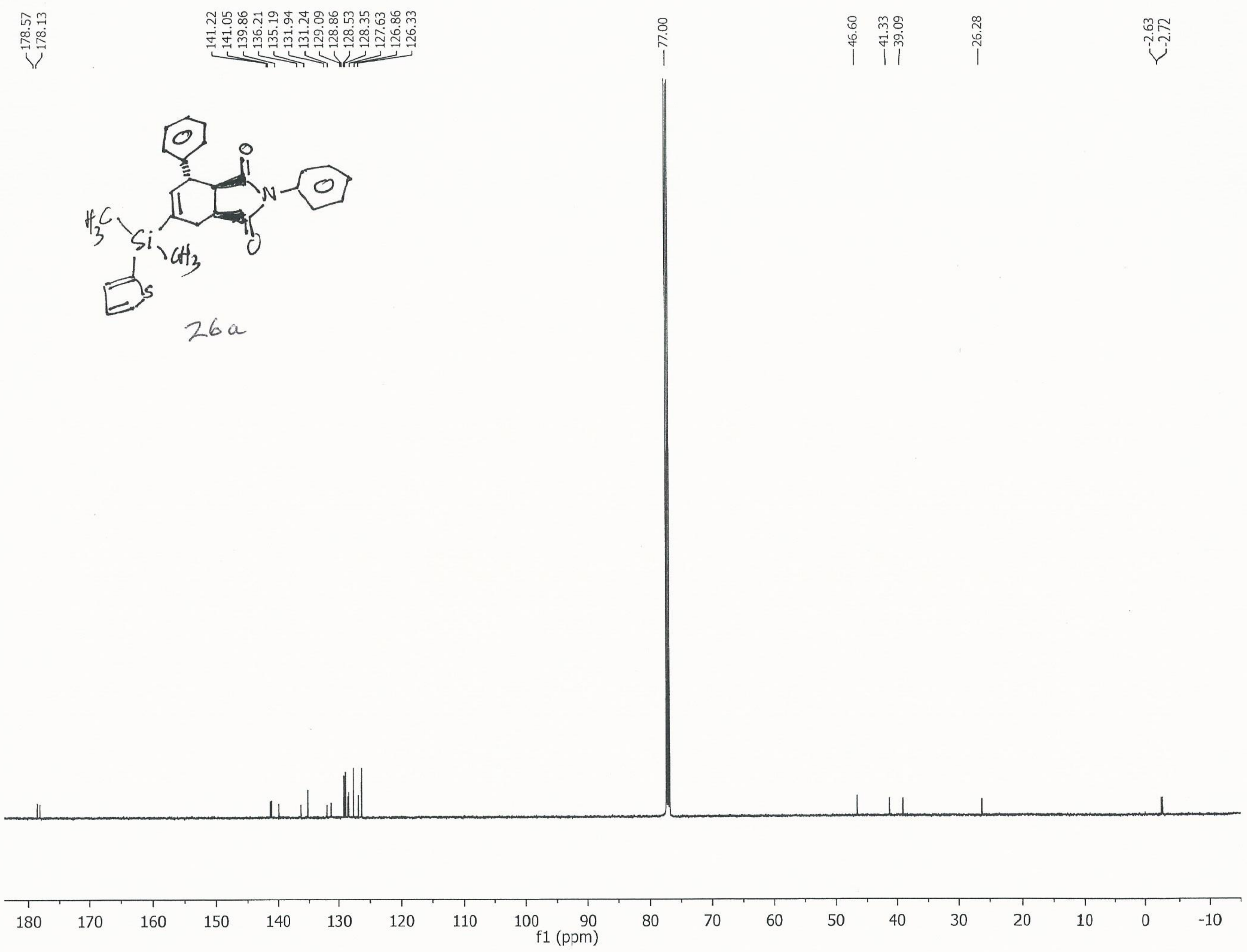

178.57
178.13
141.22
141.05
139.86
136.21
135.19
131.94
131.24
129.09
128.86
128.53
128.35
127.63
126.86
126.33
77.00
46.60
41.33
39.09
26.28
-2.63
-2.72
f1 (ppm)
26a

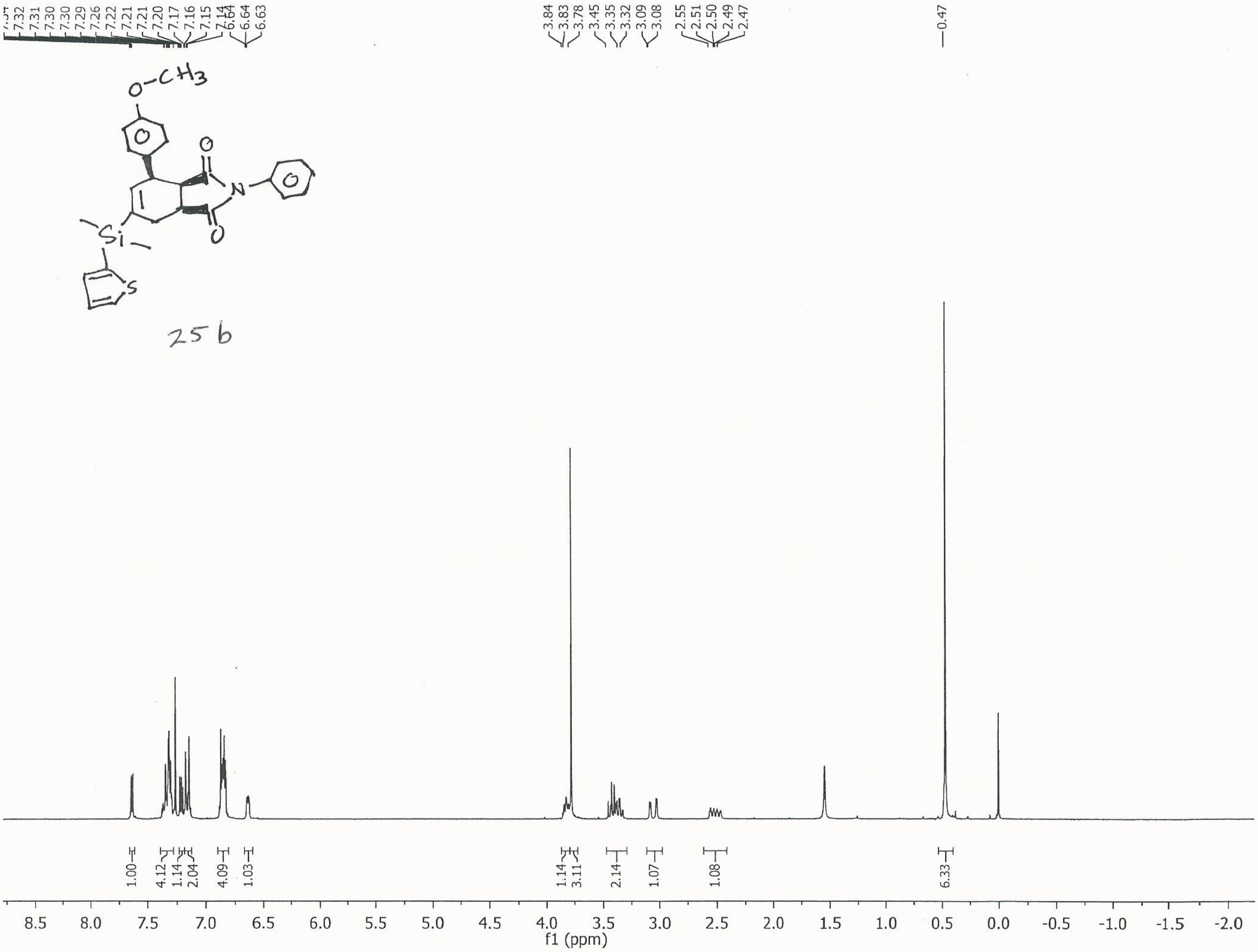
7.32
7.31
7.30
7.30
7.29
7.26
7.22
7.21
7.21
7.20
7.17
7.16
7.15
7.14
6.64
6.64
6.63
3.84
3.83
3.78
3.45
3.35
3.32
3.09
3.08
2.55
2.51
2.50
2.49
2.47
0.47
O-CH3
O
N
O
Si
S
25 b
1.00
4.12
1.14
2.04
4.09
1.03
1.14
3.11
2.14
1.07
1.08
6.33
8.5
8.0
7.5
7.0
6.5
6.0
5.5
5.0
4.5
4.0
3.5
3.0
2.5
2.0
1.5
1.0
0.5
0.0
-0.5
-1.0
-1.5
-2.0
f1 (ppm)

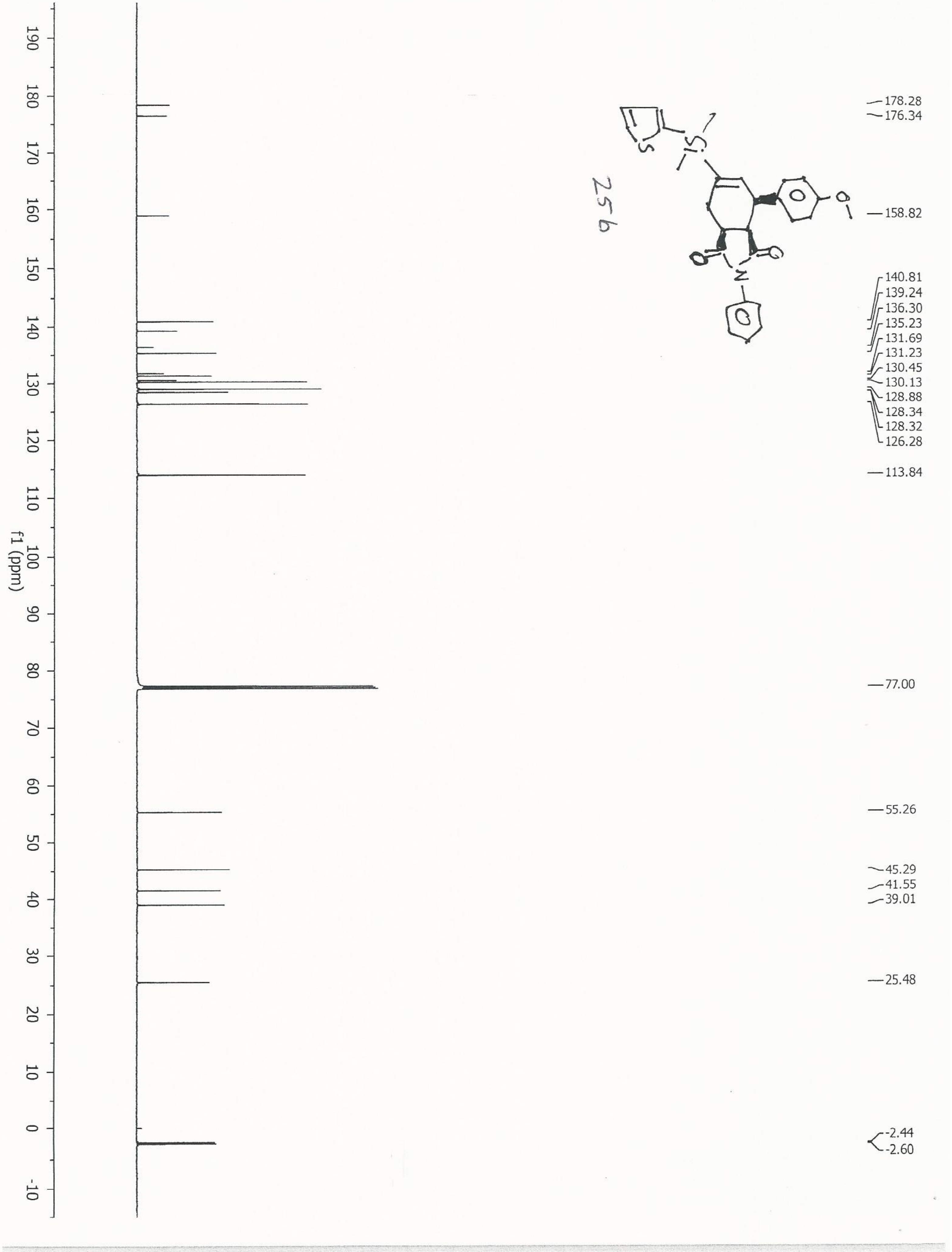
25b
178.28
176.34
158.82
140.81
139.24
136.30
135.23
131.69
131.23
130.45
130.13
128.88
128.34
128.32
126.28
113.84
77.00
55.26
45.29
41.55
39.01
25.48
-2.44
-2.60
f1 (ppm)

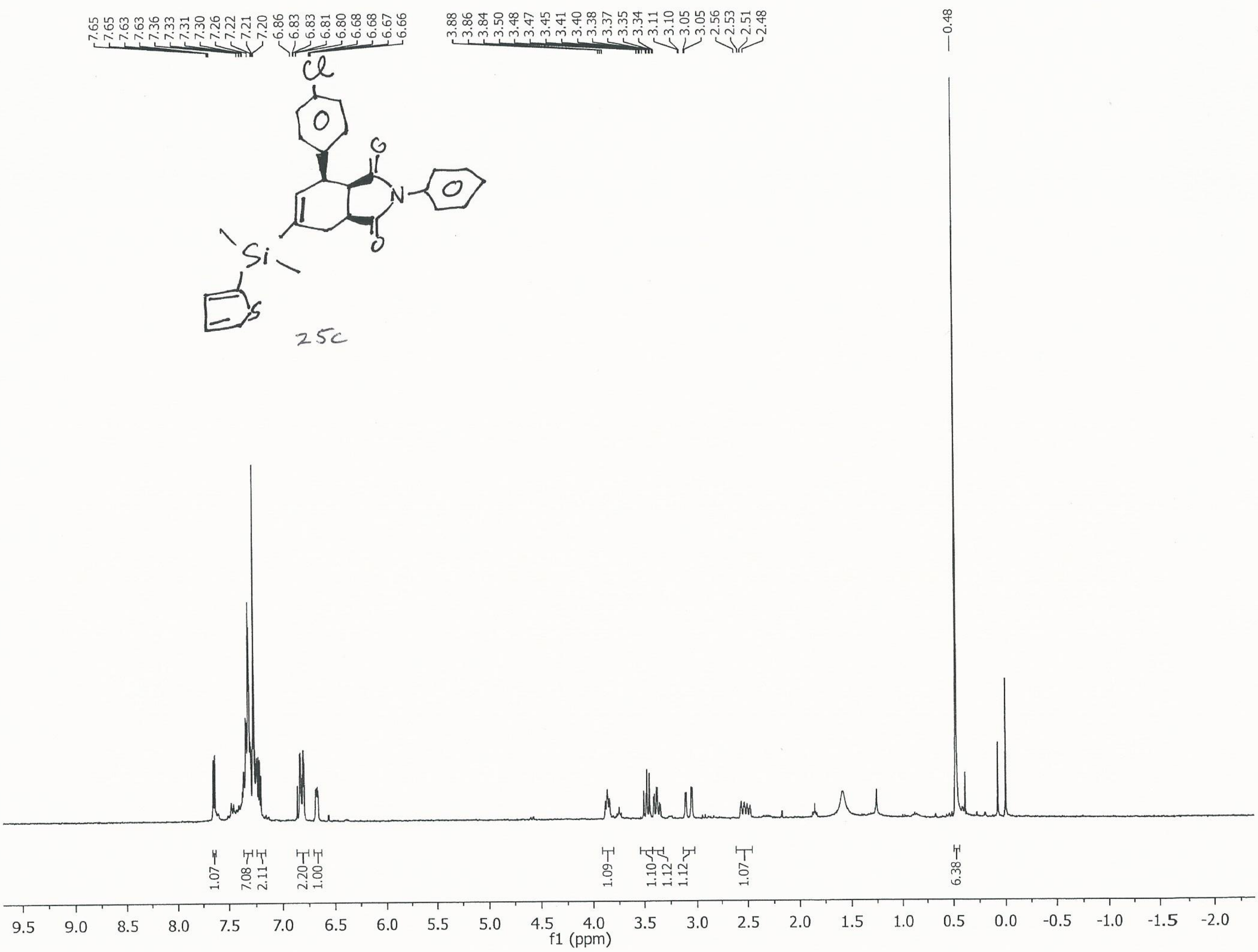
7.65
7.65
7.63
7.63
7.36
7.33
7.31
7.30
7.26
7.22
7.21
7.20
6.86
6.83
6.83
6.81
6.80
6.68
6.68
6.67
6.66
3.88
3.86
3.84
3.50
3.48
3.47
3.45
3.41
3.40
3.38
3.37
3.35
3.34
3.11
3.10
3.05
3.05
2.56
2.53
2.51
2.48
0.48
Cl
O
N
Si
S
25c
1.07
7.08
2.11
2.20
1.00
1.09
1.10
1.12
1.12
1.07
6.38
9.5
9.0
8.5
8.0
7.5
7.0
6.5
6.0
5.5
5.0
4.5
4.0
3.5
3.0
2.5
2.0
1.5
1.0
0.5
0.0
-0.5
-1.0
-1.5
-2.0
f1 (ppm)

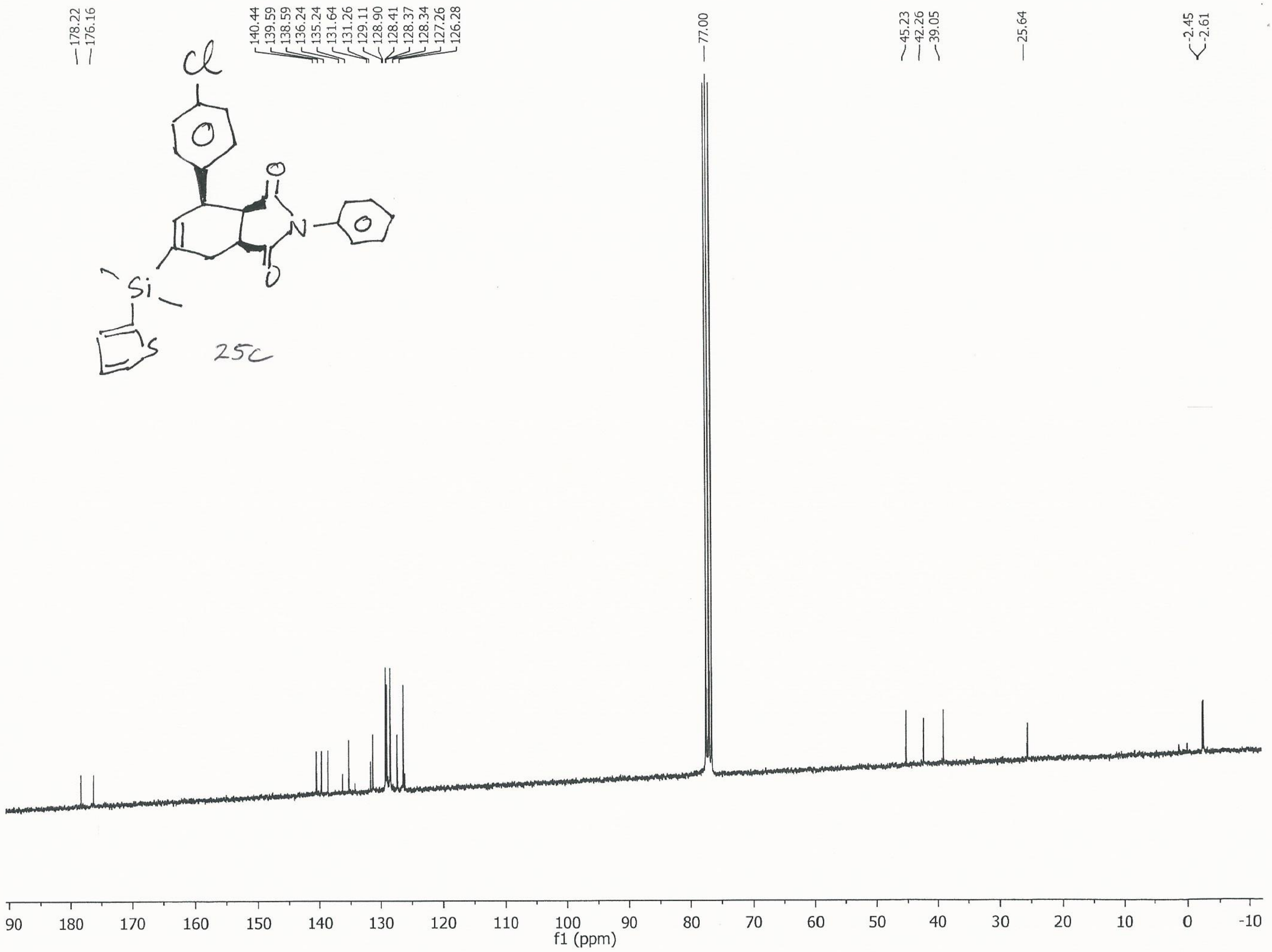

178.22
176.16
140.44
139.59
138.59
136.24
135.24
131.64
131.26
129.11
128.90
128.41
128.37
128.34
127.26
126.28
77.00
45.23
42.26
39.05
25.64
-2.45
-2.61
Cl
O
N
Si
S
25c
90
180
170
160
150
140
130
120
110
100
90
80
70
60
50
40
30
20
10
0
-10
f1 (ppm)

Superimposed NOESY and COSY spectra of Molecule 25a

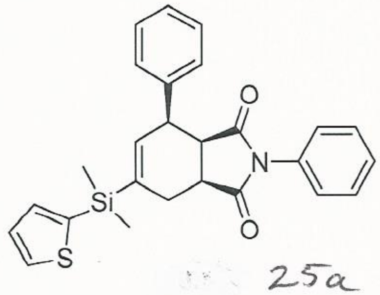

25a

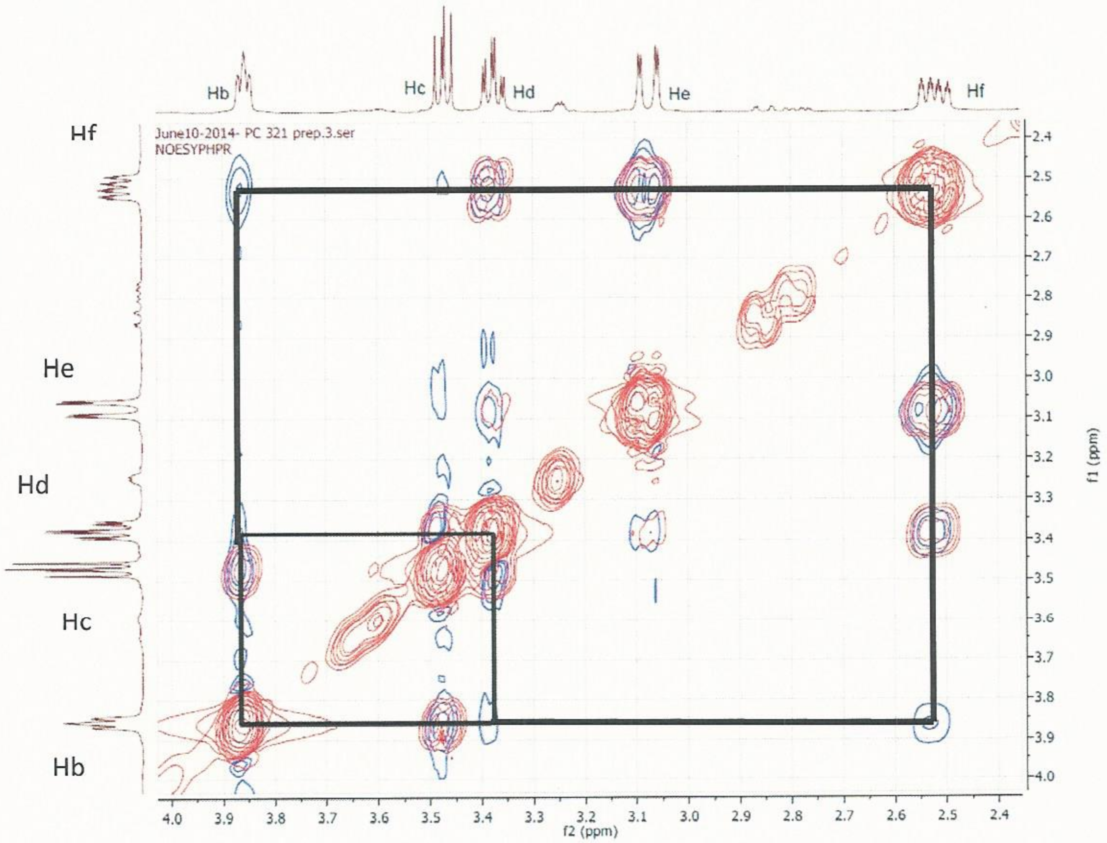

Hb
Hc
Hd
He
Hf
June10-2014- PC 321 prep.3.ser
NOESYPHPR
f2 (ppm)
f1 (ppm)

NOESY Spectrum of 25a

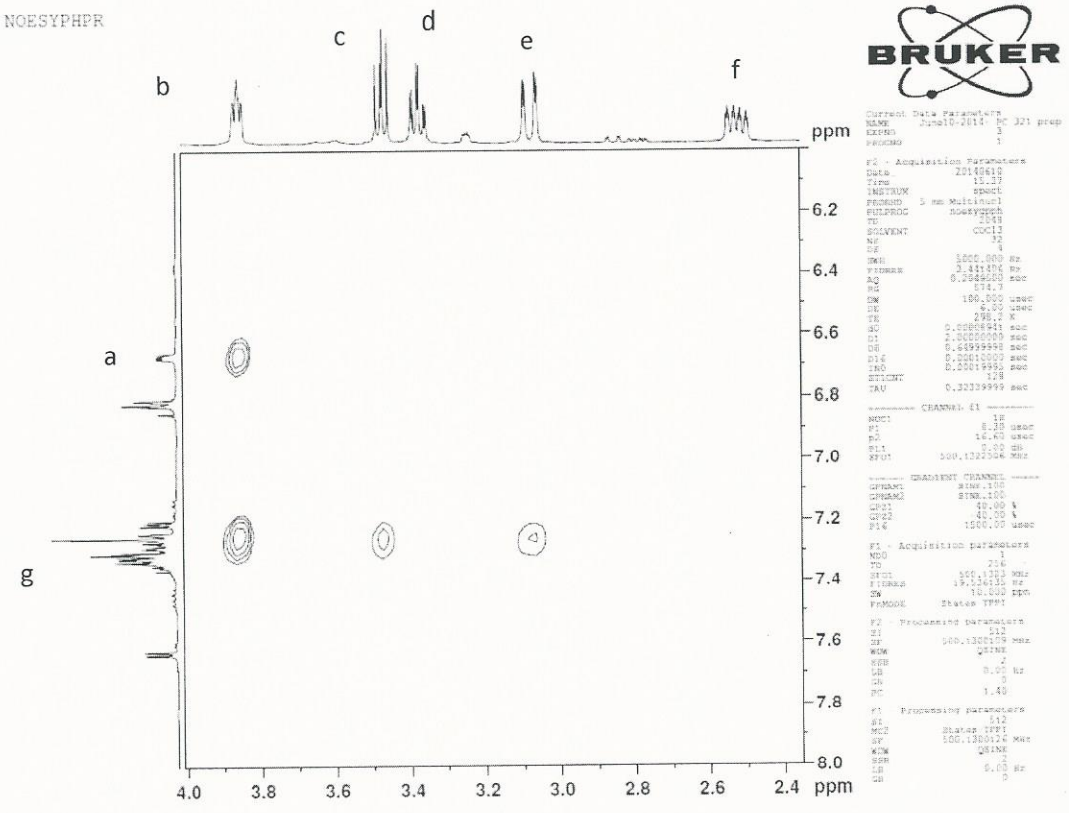
NOESYPHPR
a
b
c
d
e
f
g
ppm
6.2
6.4
6.6
6.8
7.0
7.2
7.4
7.6
7.8
8.0
4.0
3.8
3.6
3.4
3.2
3.0
2.8
2.6
2.4
ppm

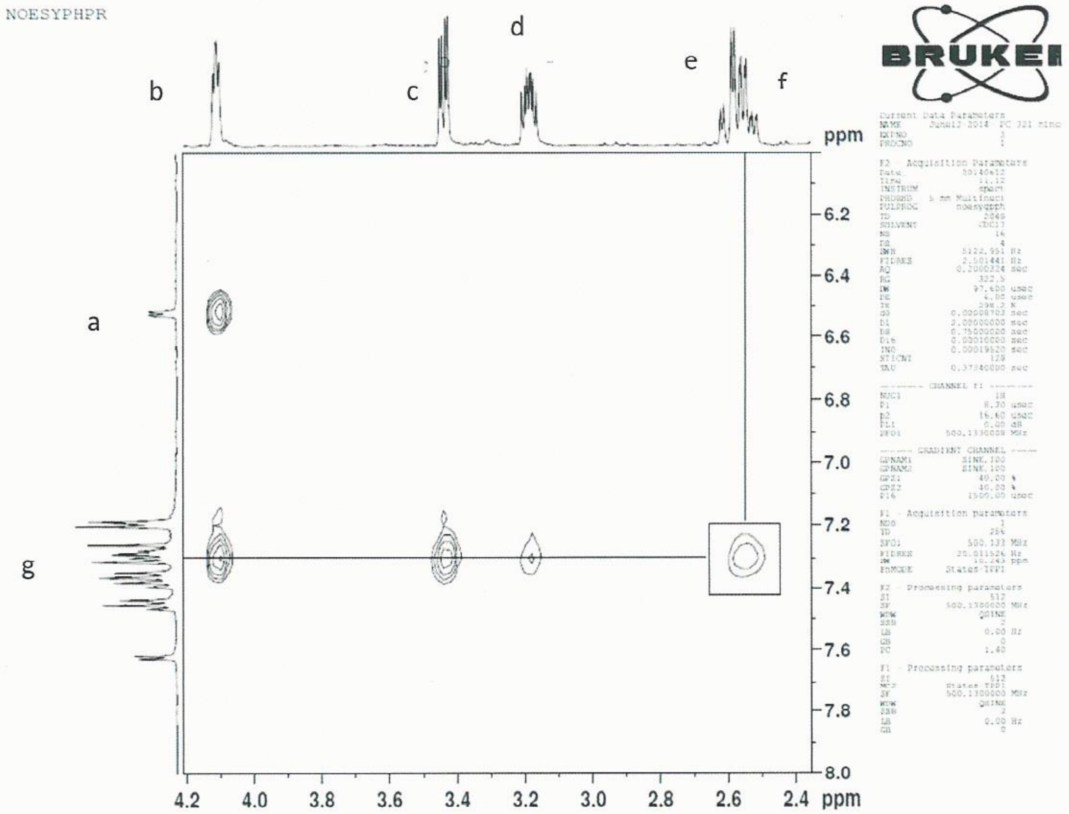
NOESYPHPR
b
c
d
e
f
a
g
ppm
6.2
6.4
6.6
6.8
7.0
7.2
7.4
7.6
7.8
8.0
4.2 4.0 3.8 3.6 3.4 3.2 3.0 2.8 2.6 2.4 ppm
Current Data Parameters
F2 - Acquisition Parameters
Time 11.12
PULPROG noesygpph
TD 2048
NS 16
DS 4
SWH 5122.951 Hz
FIDRES 2.501441 Hz
AQ 0.2000324 sec
DW 97.600 usec
D1 2.00000000 sec
D8 0.75000000 sec
D16 0.00010000 sec
IN0 0.00019520 sec
TAU 0.37340000 sec
CHANNEL f1
NUC1 1H
P1 8.30 usec
p2 16.60 usec
PL1 0.00 dB
SFO1 500.1330008 MHz
GRADIENT CHANNEL
GPNAM1 SINE.100
GPNAM2 SINE.100
GPZ1 40.00 %
GPZ2 40.00 %
P16 1500.00 usec
F1 - Acquisition parameters
TD 256
SFO1 500.133 MHz
FIDRES 20.011526 Hz
FnMODE States-TPPI
F2 - Processing parameters
SI 512
SF 500.1300000 MHz
WDW QSINE
LB 0.00 Hz
GB 0
PC 1.40
F1 - Processing parameters
SI 512
SF 500.1300000 MHz
WDW QSINE
LB 0.00 Hz
GB 0

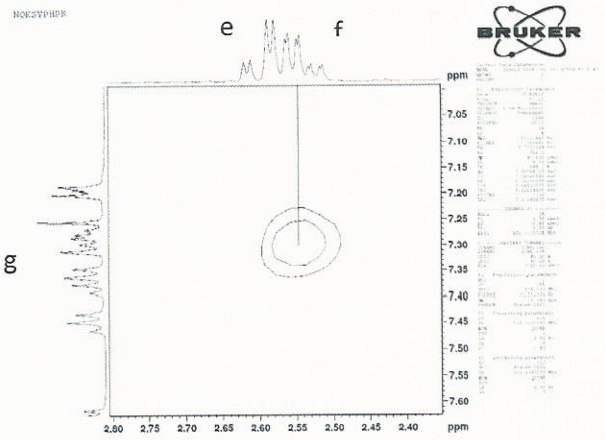
NOESYPHPR
e
f
g
ppm
7.05
7.10
7.15
7.20
7.25
7.30
7.35
7.40
7.45
7.50
7.55
7.60
2.80
2.75
2.70
2.65
2.60
2.55
2.50
2.45
2.40
ppm
